# Supplementary figures and images for: IL-25–induced shifts in macrophage polarization promote development of beige fat and improve metabolic homeostasis in mice
Source: PLoS Biol. 2021 Aug 5;19(8):e3001348. doi: 10.1371/journal.pbio.3001348 (PMC8341513; doi:10.1371/journal.pbio.3001348)

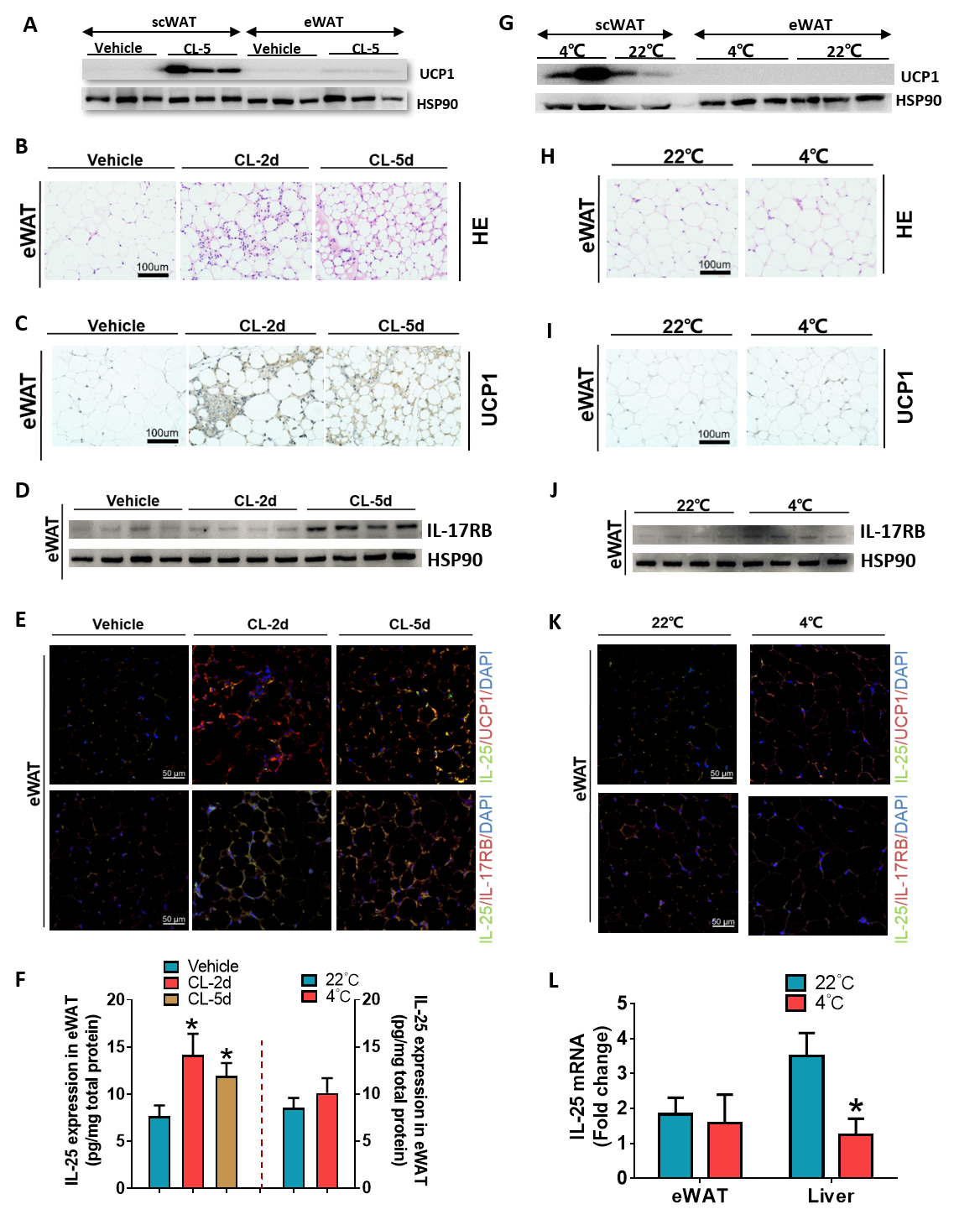

Supplement: S1 Fig — (A–F) WT mice were injected with CL (1 mg/kg body weight) for 2 (CL-2d) and 5 days (CL-5d). (A) The protein level of UCP1 was analyzed by western blot in WAT. HSP90 was used as a loading control. (B) H&E staining of eWAT. 400× magnification. (C) Immunohistochemical staining for UCP1 of eWAT. 400× magnification. (D) The protein level of IL-17RB was analyzed by western blot in eWAT. HSP90 was used as a loading control. (E) Immunofluorescent staining for IL-25 (IL-25+ green) and UCP1 (UCP1+ red) or IL-17RB (IL-17RB+ red) of eWAT. Nucleus stained with DAPI (blue). Images were photographed at 200× magnification. (F) IL-25 protein expression in eWAT (n = 4–5 per treatment). (G–L) WT mice were placed at 22°C or 4°C in individual cages for 48 hours (n = 4–5 per treatment). (G) The protein level of UCP1 was analyzed by western blot in WAT. HSP90 was used as a loading control. (H) H&E staining of eWAT in 22°C or 4°C for 48 hours. 400× magnification. (I) Immunohistochemical staining for UCP1 of eWAT at 22°C or 4°C for 48 hours. 400× magnification. (J) The protein level of IL-17RB was analyzed by western blot of eWAT at 22°C or 4°C for 48 hours. HSP90 was used as a loading control. (K) Immunofluorescent staining for IL-25 (IL-25+ green) and UCP1 (UCP1+ red) or IL-17RB (IL-17RB+ red) of eWAT at 22°C or 4°C for 48 hours. Nucleus stained with DAPI (blue). Images were photographed at 200× magnification. (L) Results of RT-qPCR analysis showing the expression of IL-25 in eWAT and liver of mice maintained at 22°C or 4°C for 48 hours. (n = 4–5). *p < 0.05 by two-sided unpaired t test. Data present as mean ± SEM. The data underlying this figure can be found in S1 Data. CL, CL-316, 243; eWAT, epididymal WAT; H&E, hematoxylin and eosin; IL, interleukin; IL-17RB, IL-17 receptor B; RT-qPCR, Reverse transcription - quantitative PCR; scWAT, subcutaneous WAT; UCP1, uncoupling protein 1;WAT, white adipose tissue; WT, wild-type. (TIF) [file pbio.3001348.s001.tif]

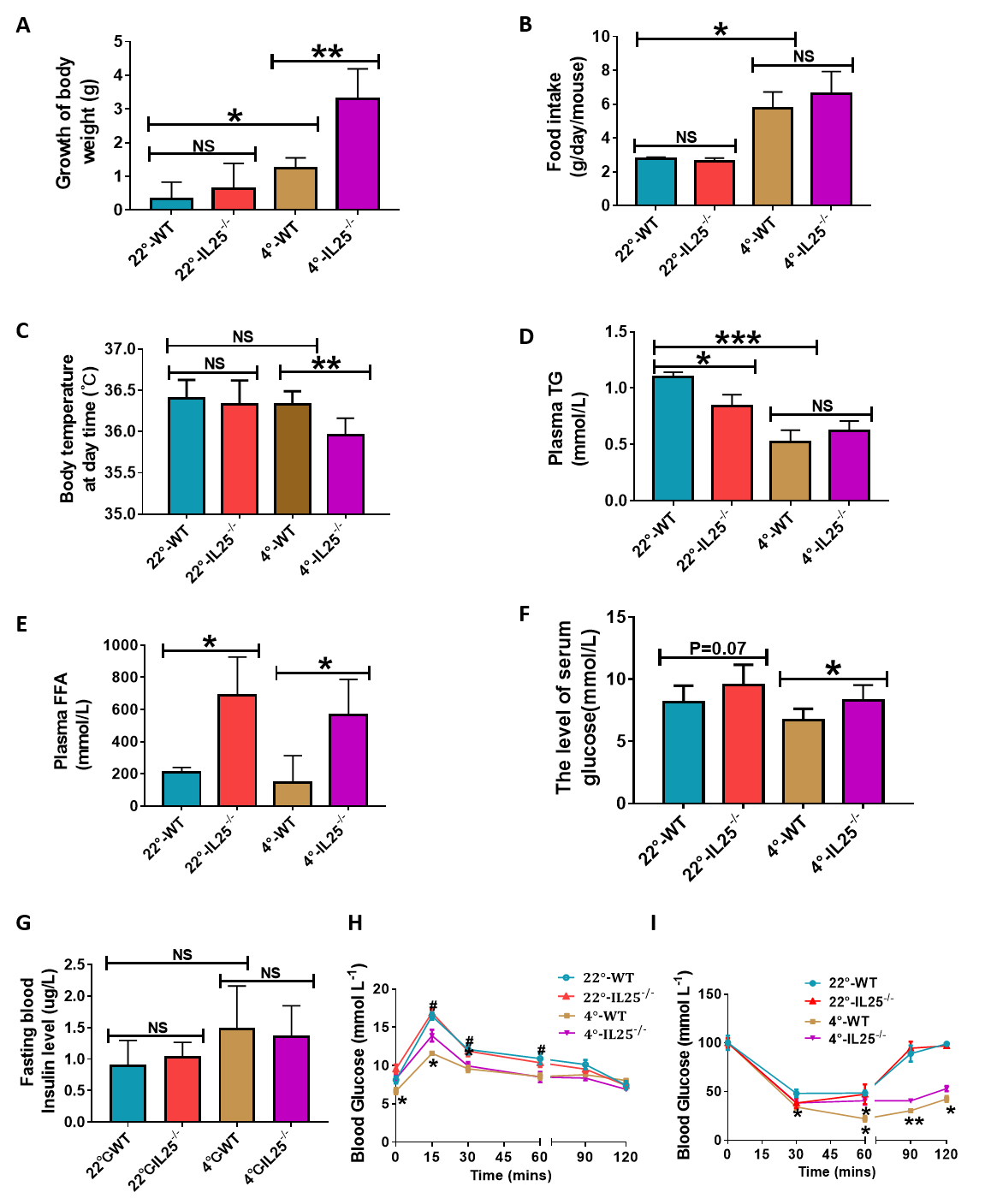

Supplement: S2 Fig — WT and IL-25−/− mice were placed at 22°C or 4°C for 48 hours. (A) Changes in body mass of mice. (B) The daily food intake. (C) The temperature of mice after treatment. (D) The TG level and (E) the FFA level of mice after treatment. (F) Changes in fasting blood. (G) Changes of fasting insulin level in mice. (H) GTT. (I) ITT was conducted by IP injection of glucose (1 g·kg−1) or injection of insulin (1 U·kg−1) and measurement of blood glucose concentration in overnight-fasted mice. The tissues were harvested within 10 minutes after an injection of insulin (1 U·kg−1). *p < 0.05, compared with control group by two-sided unpaired t test. Data present as mean ± SEM. The data underlying this figure can be found in S1 Data. FFA, free fatty acid; acid; GTT, glucose tolerance test; IL, interleukin; IP, intraperitoneal; ITT, insulin tolerance test; NS, not significant; TG, triglyceride; WT, wild-type. (TIF) [file pbio.3001348.s002.tif]

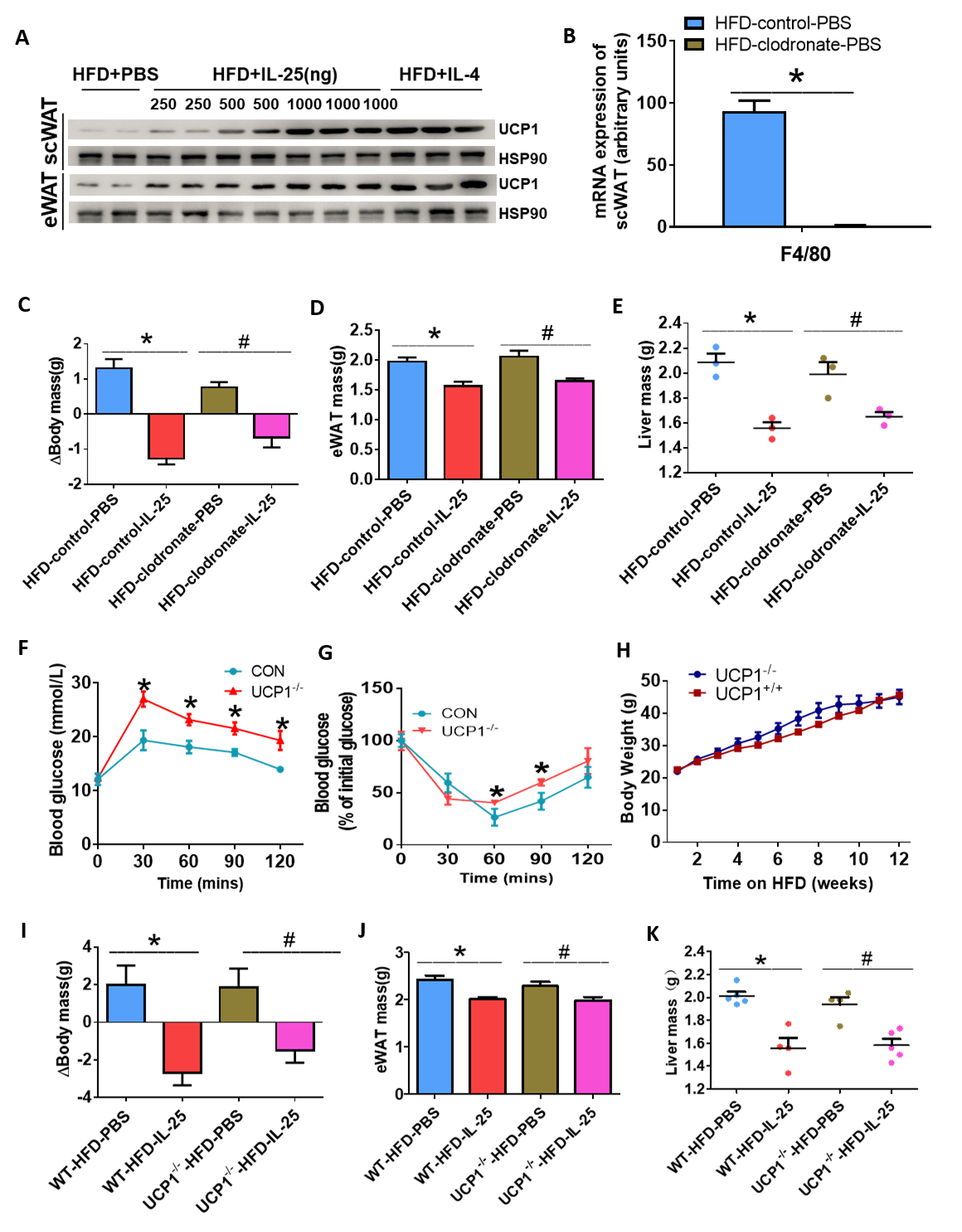

Supplement: S3 Fig — (A) The western blot analysis level of UCP1 protein in scWAT and eWAT from mice injected with vehicle and various doses of IL-25 for 14 days. (B) Depletion of macrophage with clodronate and the mRNA expression of F4/80 examined by RT-qPCR in scWAT. (C–E) DIO mice (n = 5) administrated with clodronate-loaded liposomes to obliterate macrophages and then injected with vehicle or IL-25 (1 μg/day) for 14 days. Changes in body mass (C), eWAT mass (D), and liver mass (E) from different treatment groups. (F–K) WT (UCP1+/+) and UCP1-null (UCP1−/−) mice (n = 5 per treatment) were fed with an HFD for 12 weeks and then injected with IL-25 (1 μg) or vehicle for 14 days. (F) GTT was conducted by oral glucose (2 g·kg−1) and measurement of blood glucose concentration with OneTouch Ultra Glucometer at designed time points in overnight-fasted mice. (G) ITT was done by IP injection of insulin (0.75 U·kg−1) and measurement of blood glucose concentration by OneTouch Ultra Glucometer at designed time points in 8 h–fasted mice. (H) Weight of WT (UCP1+/+) and UCP1-null (UCP1−/−) mice assessed after feeding HFD. (I) Changes in body mass. (J) eWAT mass. (K) Liver mass from different treatment groups. #p < 0.05, compared with UCP1−/−-HFD-PBS group, *p < 0.05, compared with WT-HFD-PBS group by two-sided unpaired t test. Data present as mean ± SEM. The data underlying this figure can be found in S1 Data. DIO, diet-induced obesity; eWAT, epididymal WAT; GTT, glucose tolerance test; HFD, high-fat diet; IL, interleukin; IP, intraperitoneal; ITT, insulin tolerance test; RT-qPCR, quantitative real-time PCR; scWAT, subcutaneous WAT; UCP1, uncoupling protein 1; WT, wild-type. (TIF) [file pbio.3001348.s003.tif]
