# Supplementary material for: IL-25–induced shifts in macrophage polarization promote development of beige fat and improve metabolic homeostasis in mice
Source: PLoS Biol. 2021 Aug 5;19(8):e3001348. doi: 10.1371/journal.pbio.3001348 (PMC8341513; doi:10.1371/journal.pbio.3001348)
Supplement: S1 Raw Images — (PDF) [file pbio.3001348.s005.pdf]

Fig1B

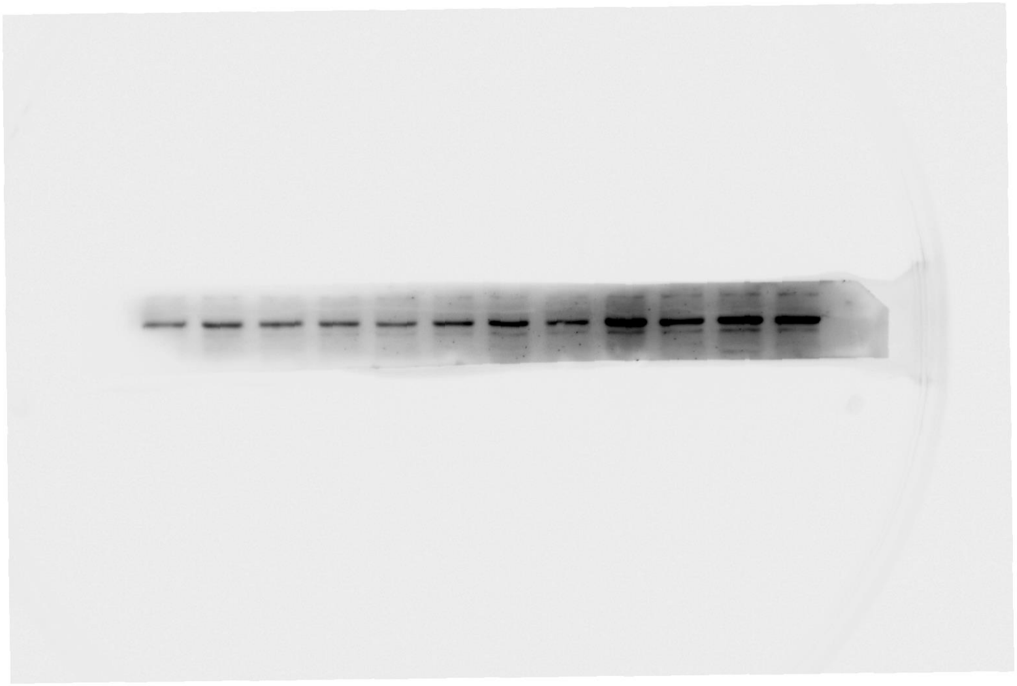

IL17RB

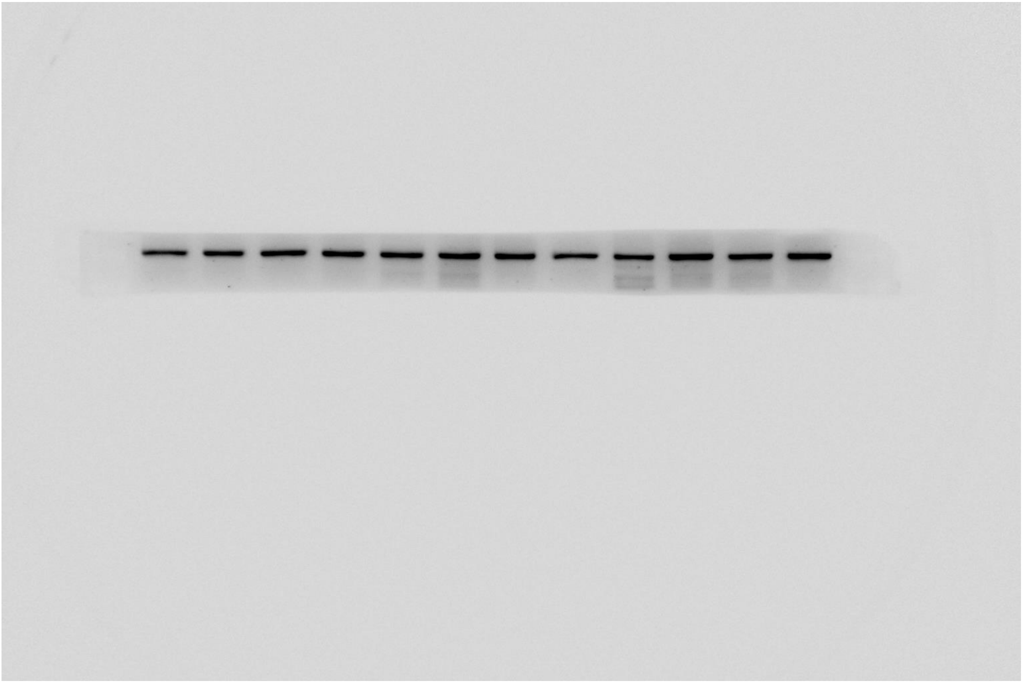

HSP90

Fig1E

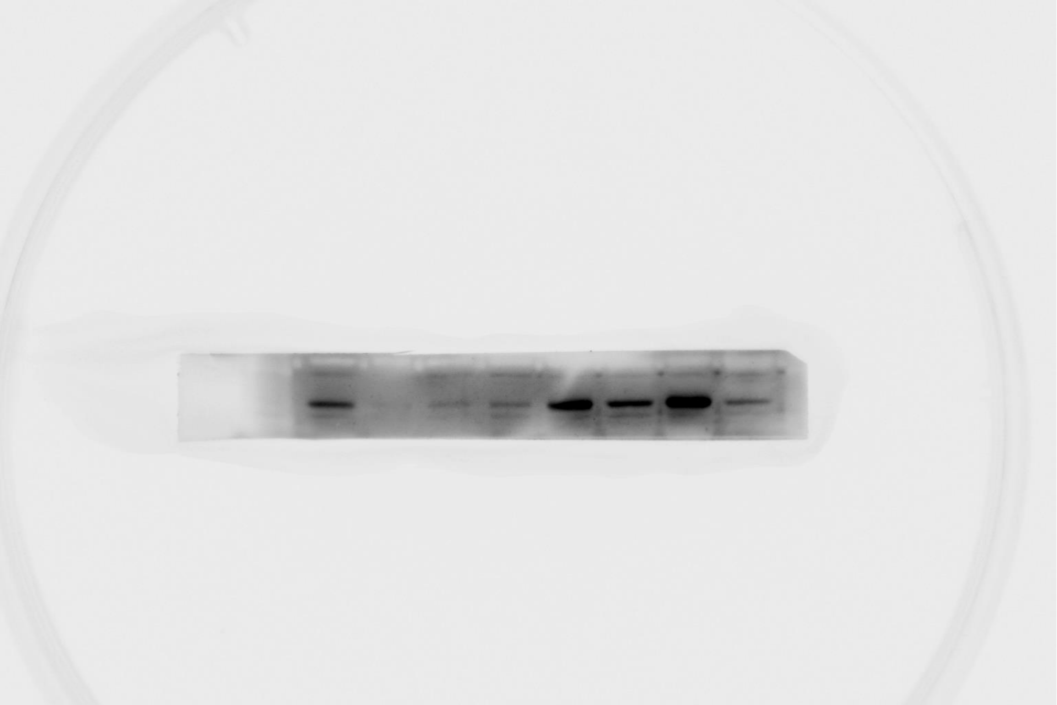

IL17RB

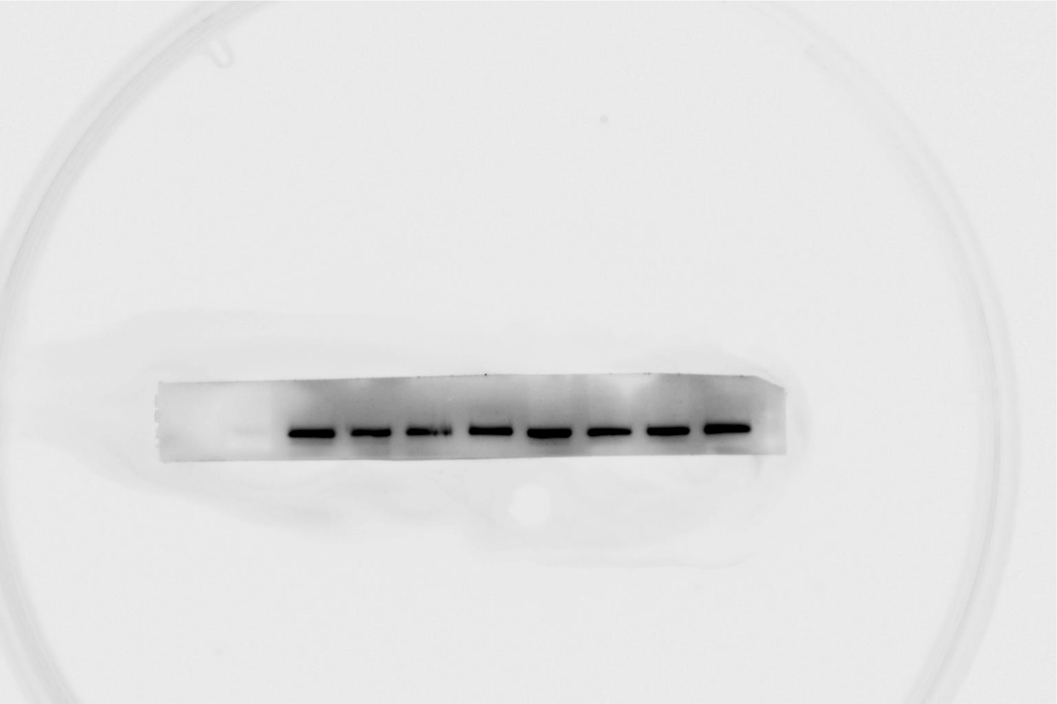

HSP90

Fig1K

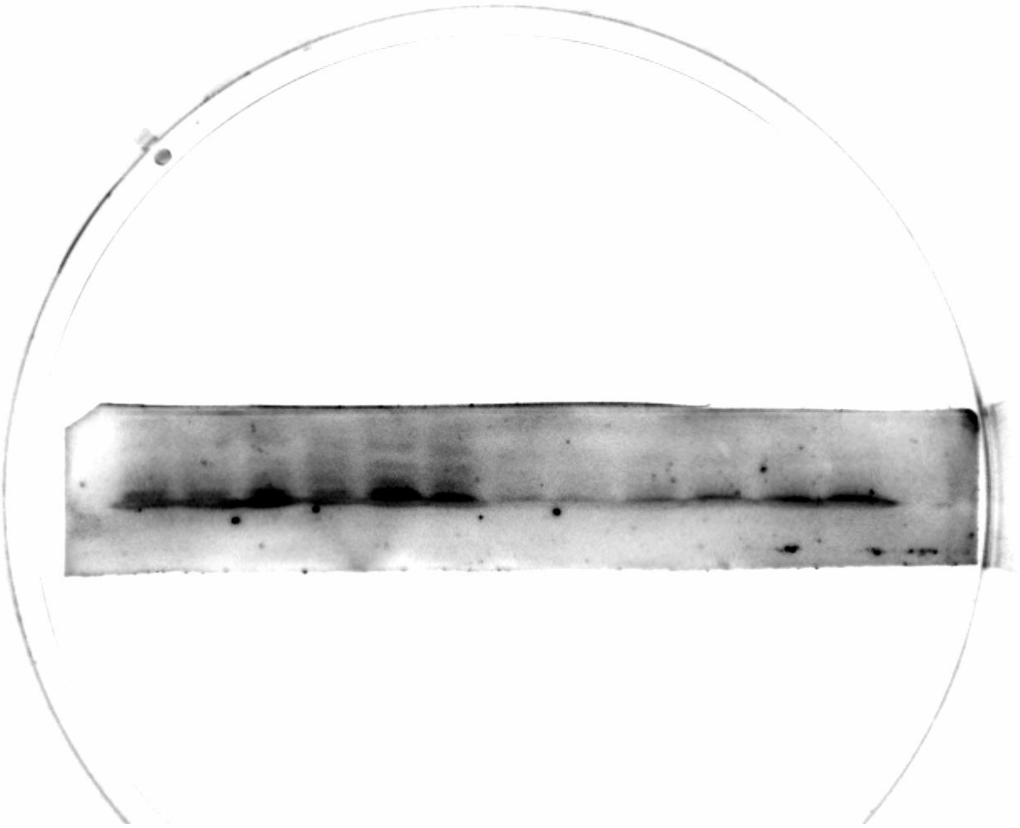

IL-25

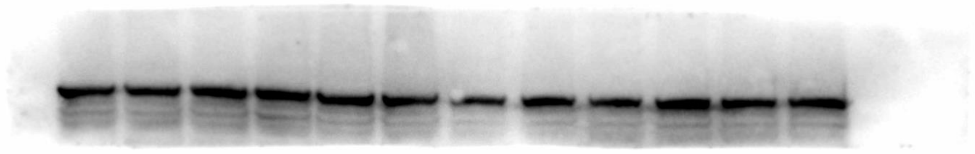

B-actin

Fig2A

scWAT-HSP90

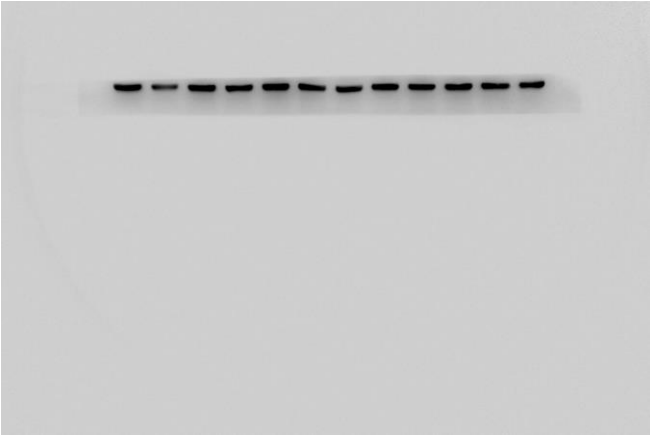

scWAT-UCP1

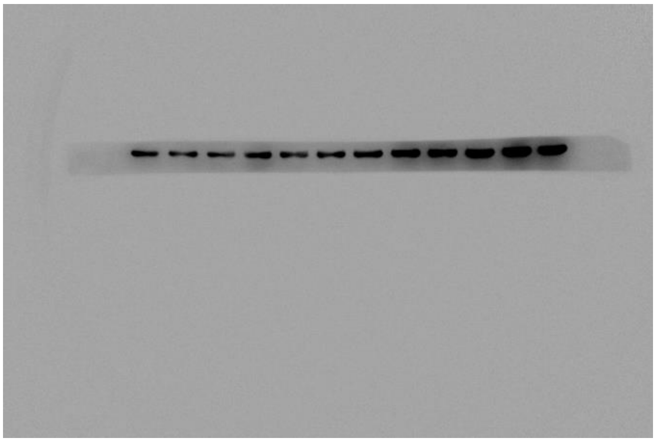

eWAT-HSP90

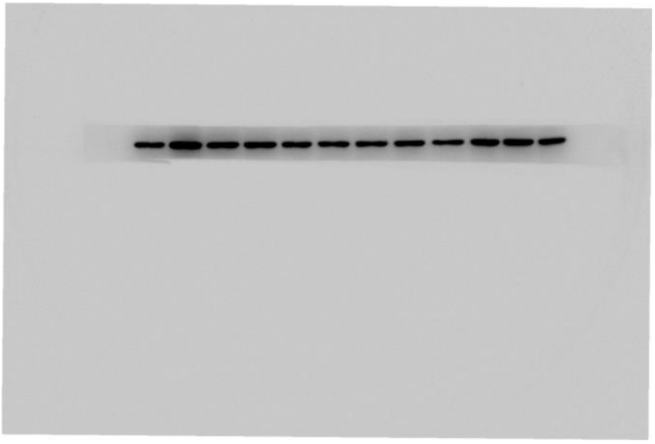

eWAT-UCP1

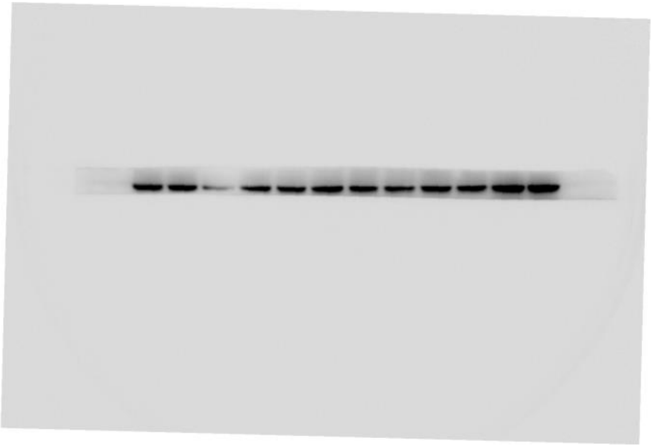

BAT-HSP90

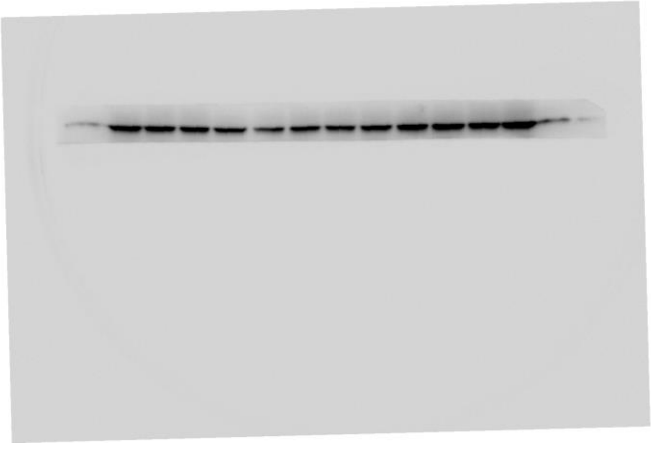

BAT-UCP1

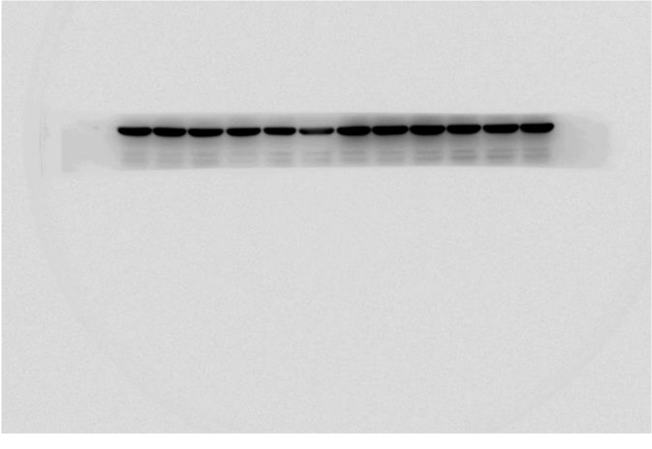

Fig2D

scWAT-IL17RB

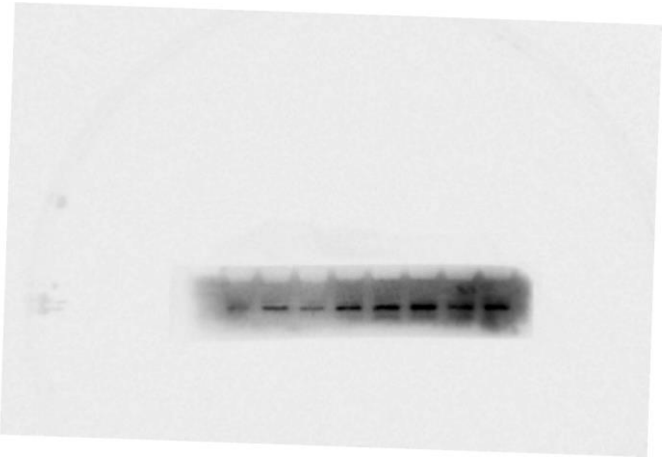

eWAT-IL17RB

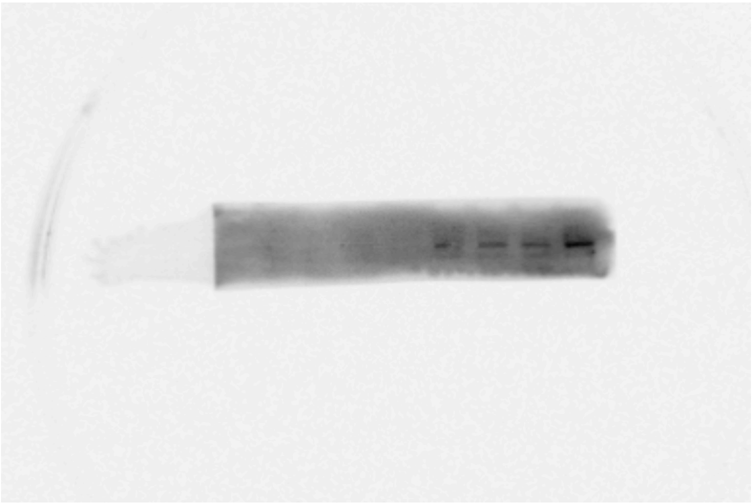

scWAT-UCP1

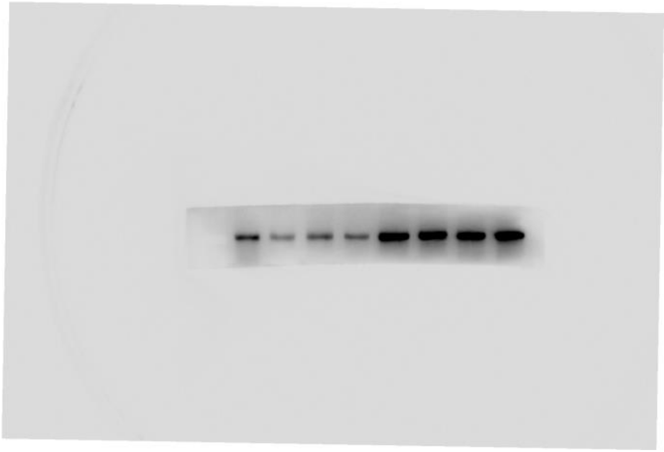

eWAT-UCP1

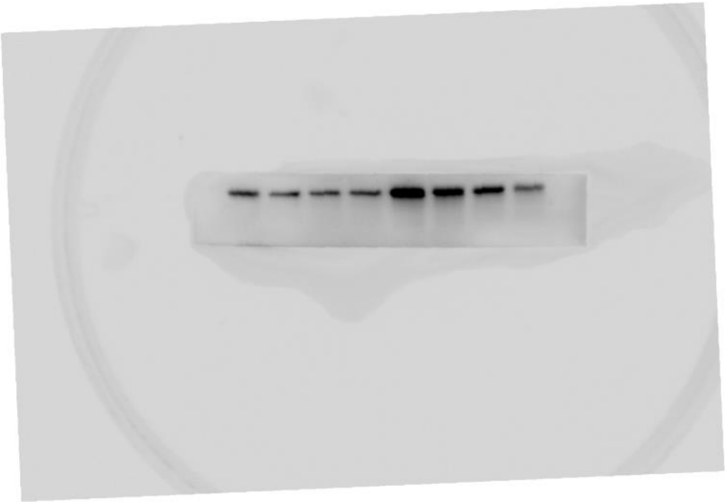

scWAT-HSP90

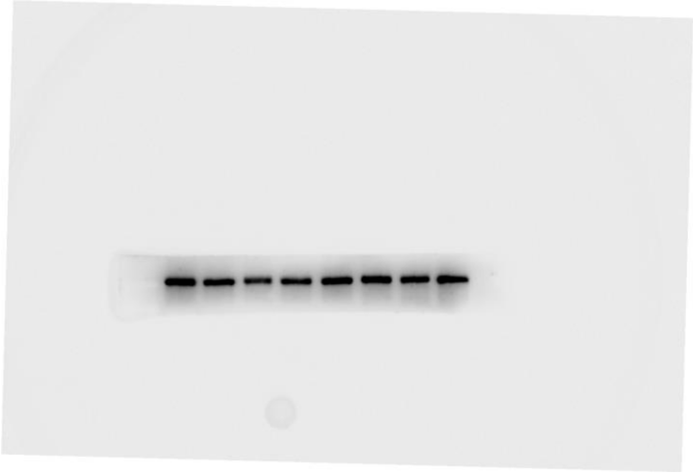

eWAT-HSP90

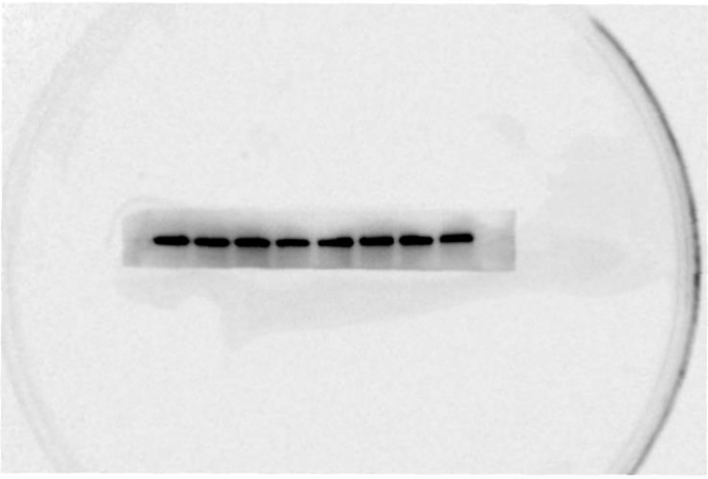

Fig2I

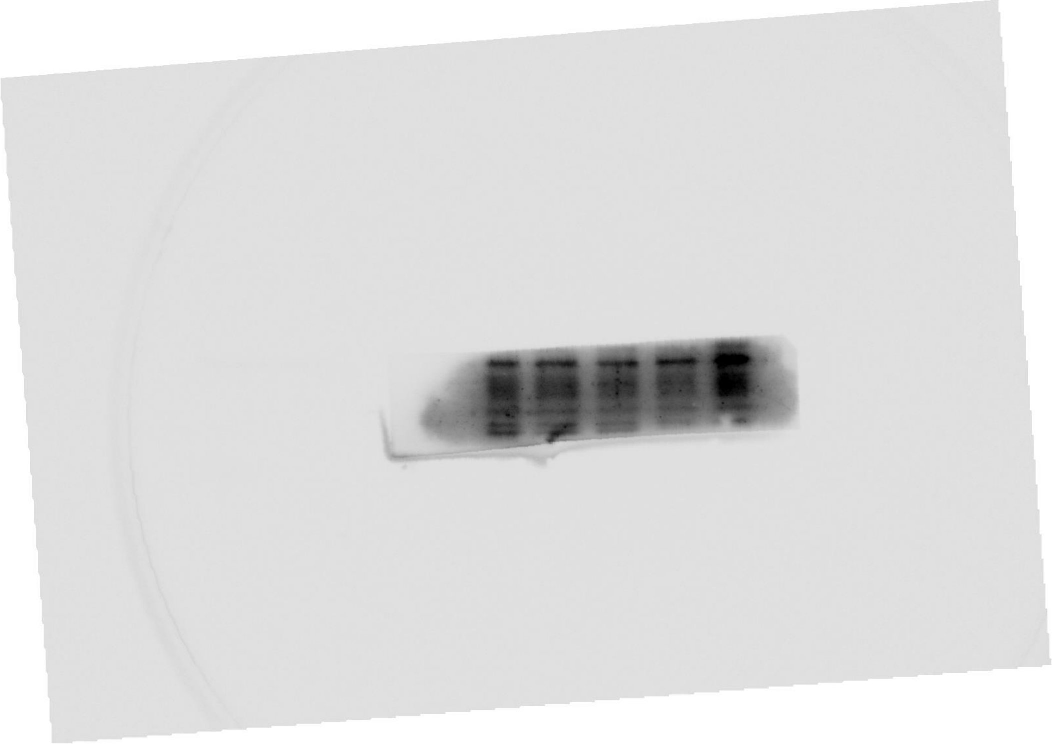

UCP1

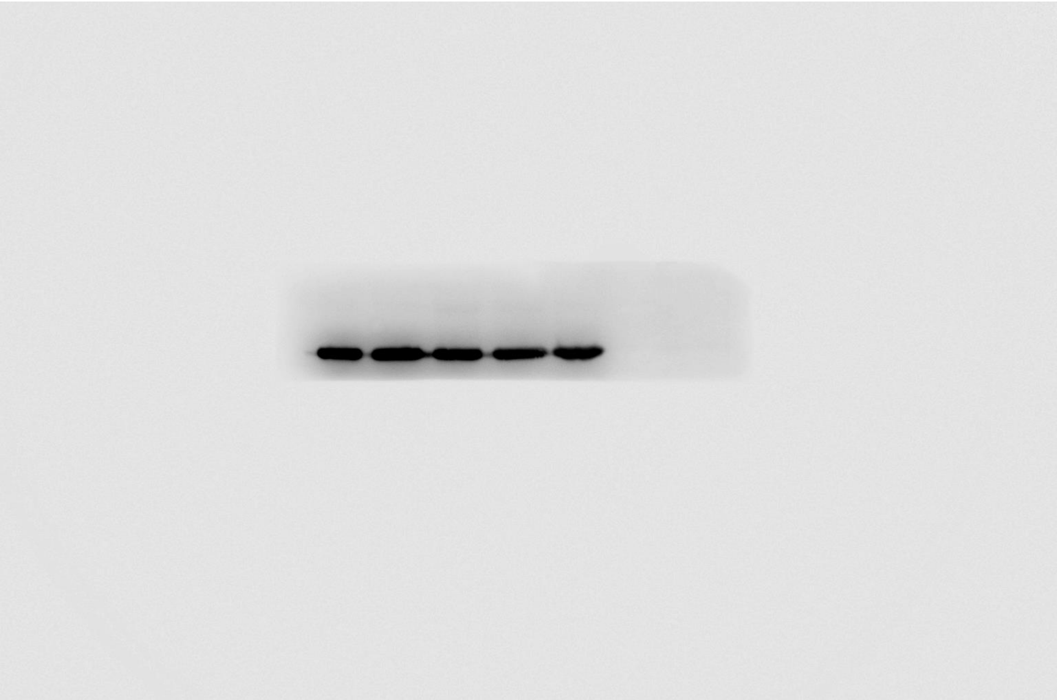

HSP90

Fig3E

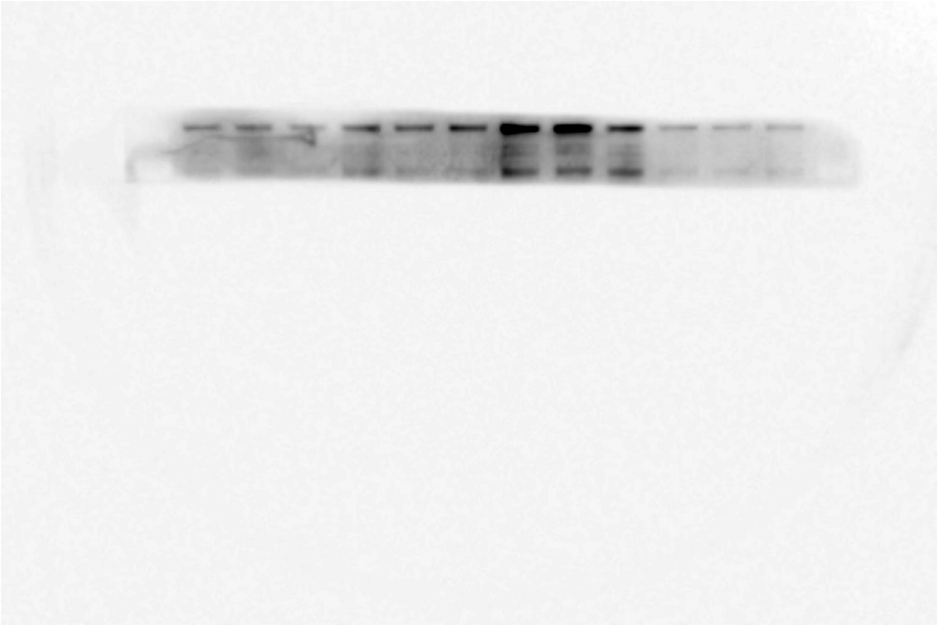

UCP1

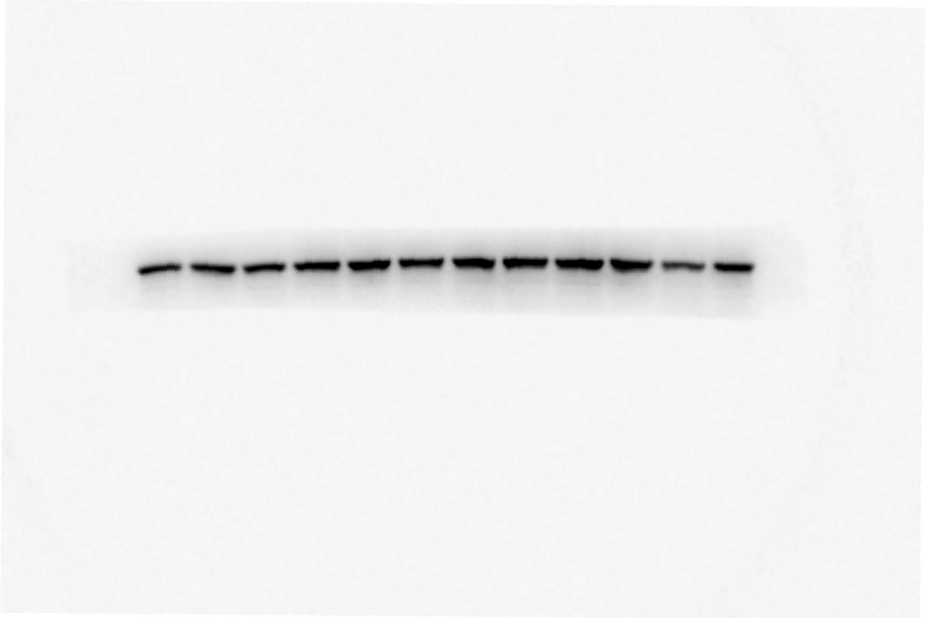

HSP90

Fig3F

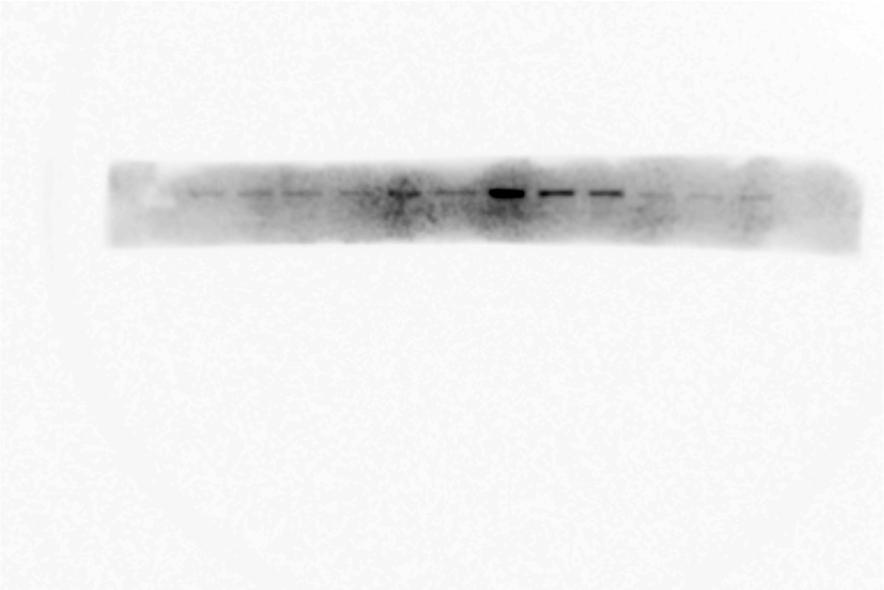

UCP1

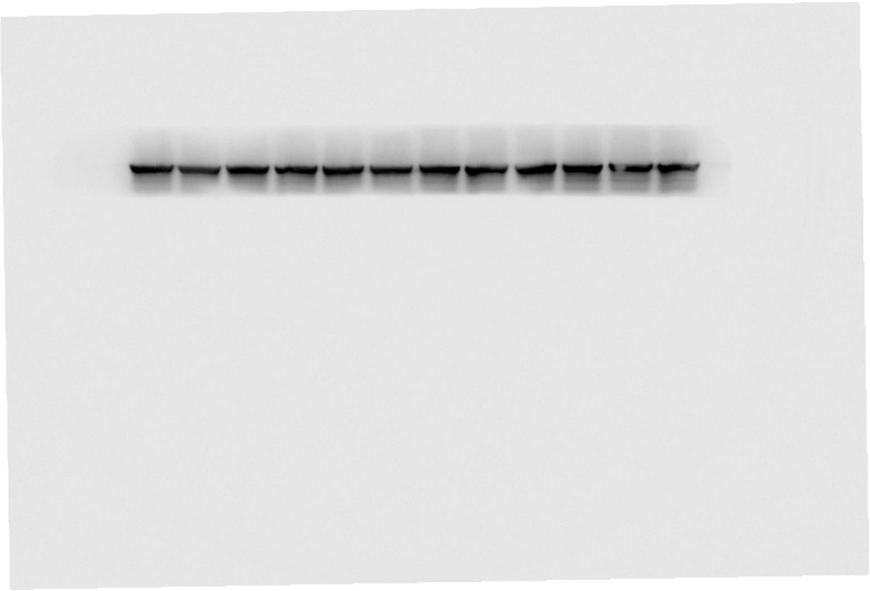

HSP90

Fig3H

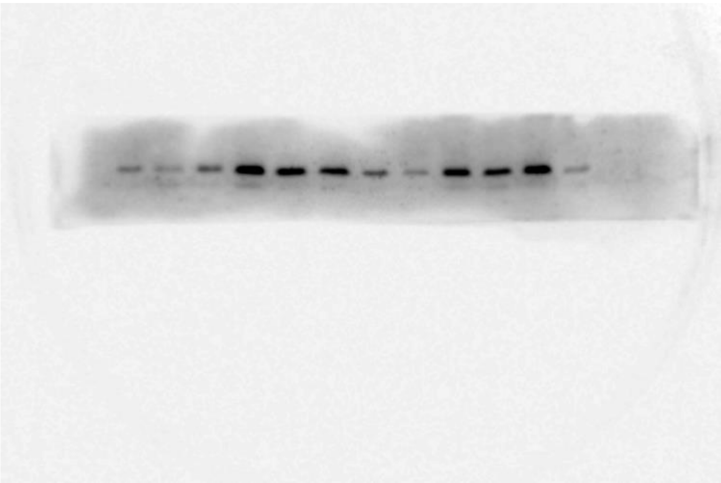

scWAT-UCP1

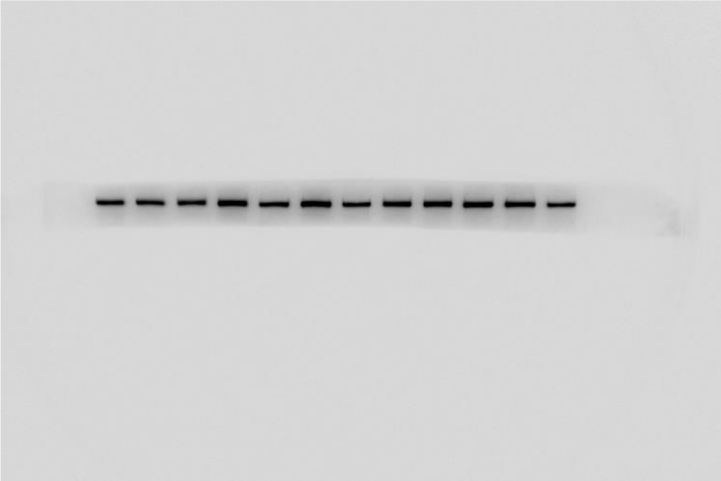

scWAT-HSP90

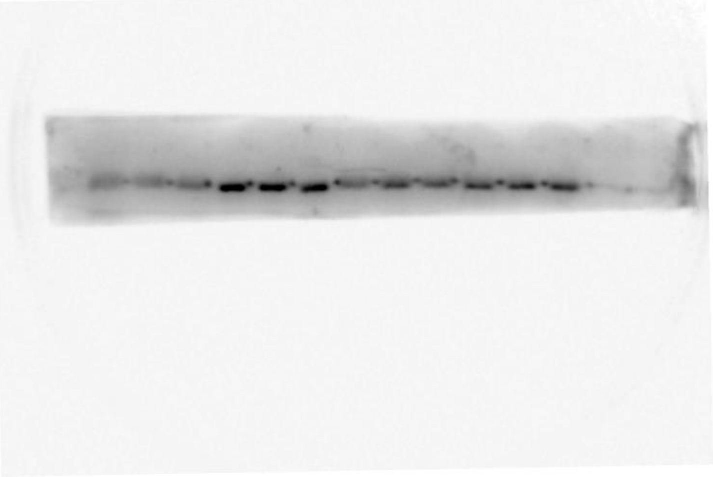

eWAT-UCP1

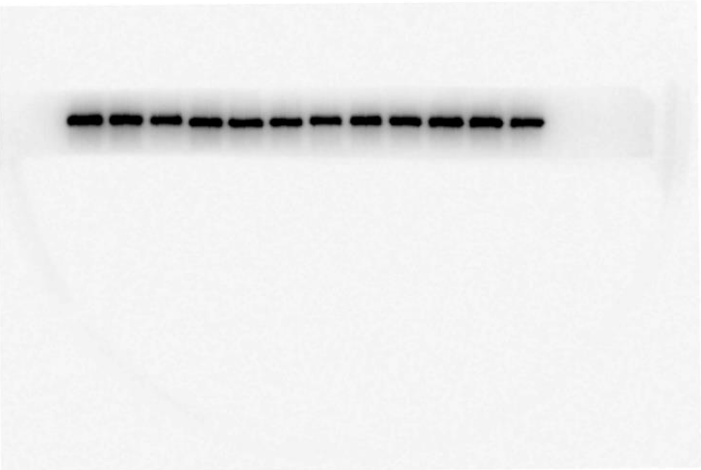

eWAT-HSP90

Fig4A

scWAT-UCP1

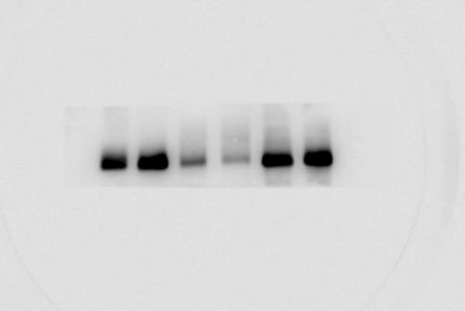

scWAT-HSP90

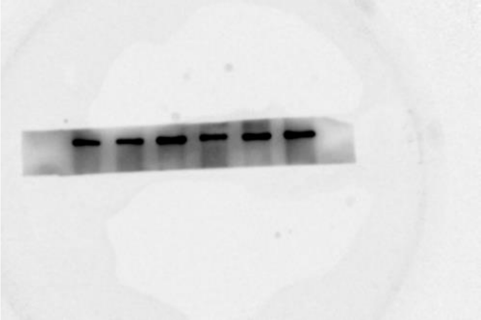

scWAT-TH

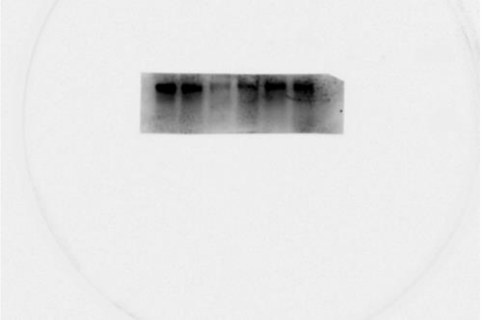

eWAT-UCP1

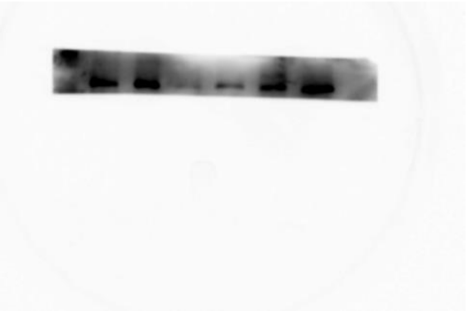

eWAT-HSP90

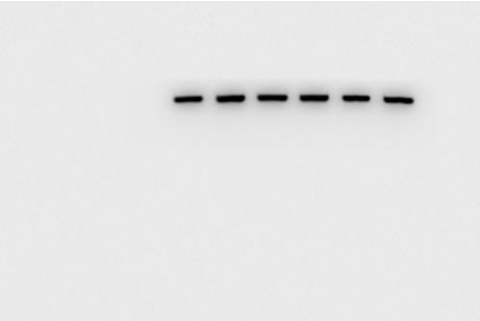

eWAT-TH

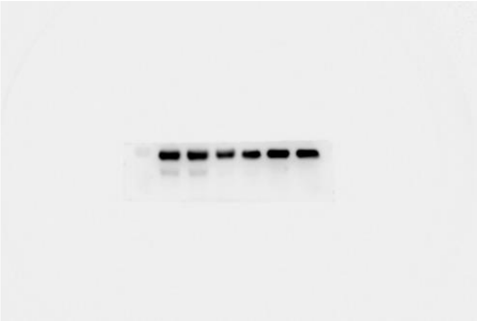

BAT-UCP1

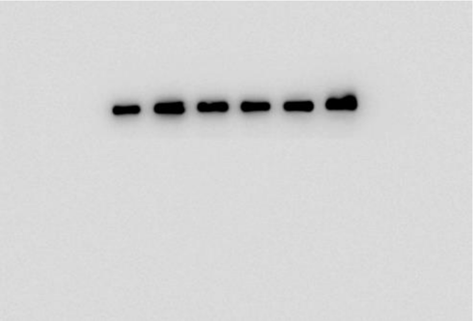

BAT-HSP90

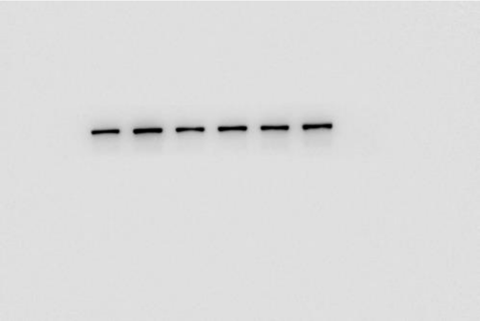

BAT-TH

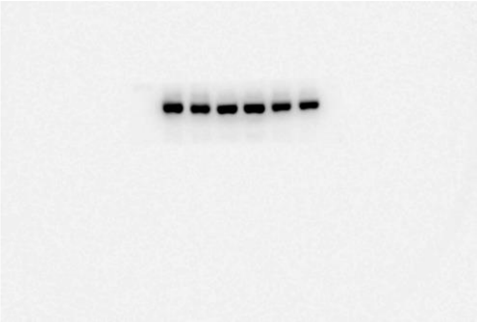

Fig4B

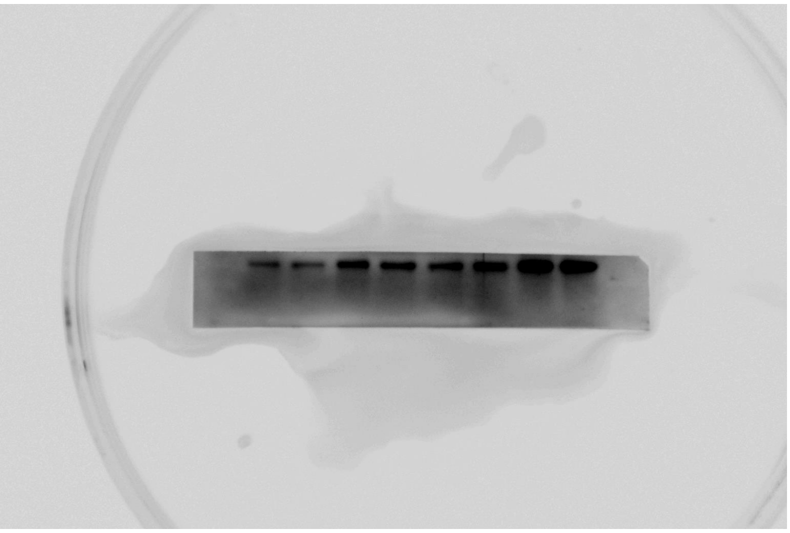

scWAT-TH

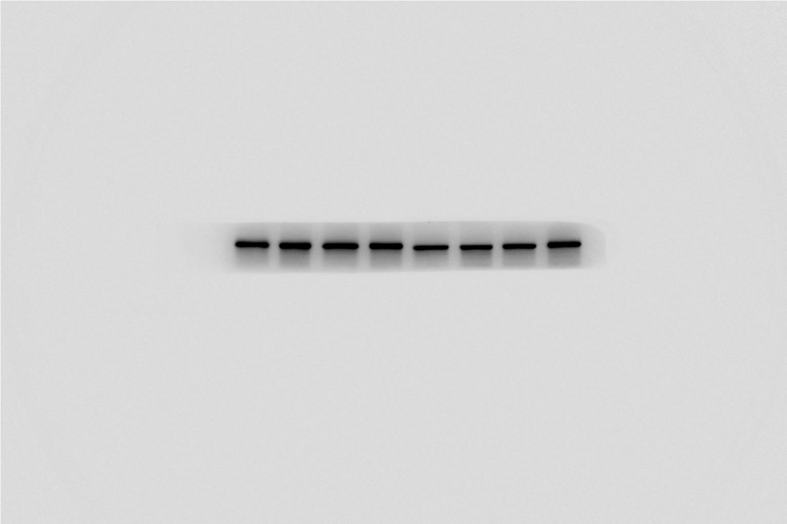

scWAT-HSP90

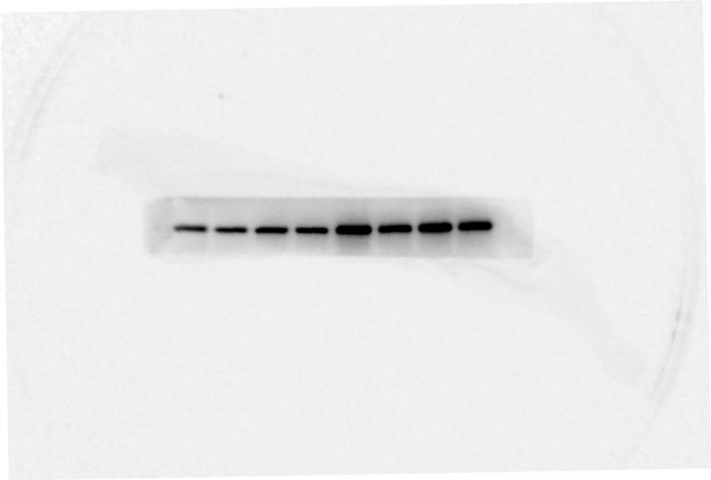

eWAT-TH

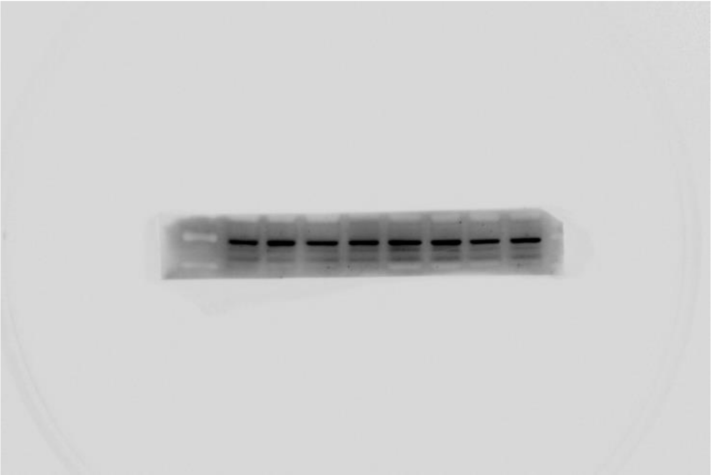

eWAT-HSP90

Fig4G

scWAT-TH

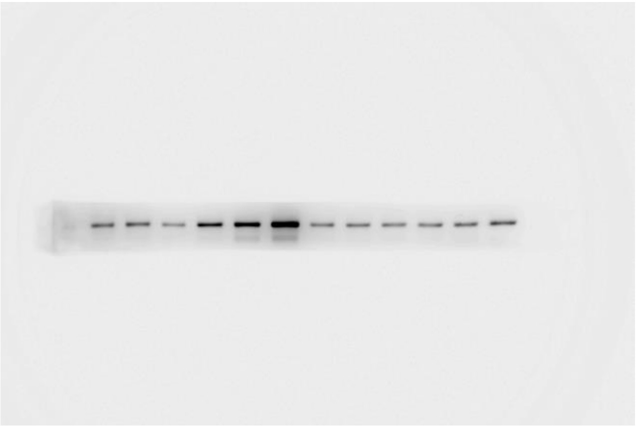

eWAT-TH

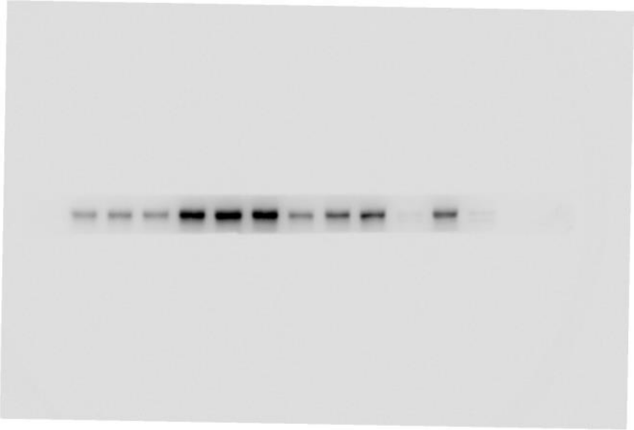

scWAT-UCP1

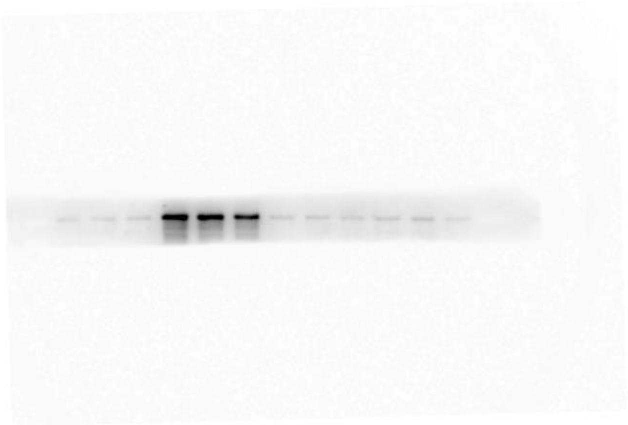

eWAT-UCP1

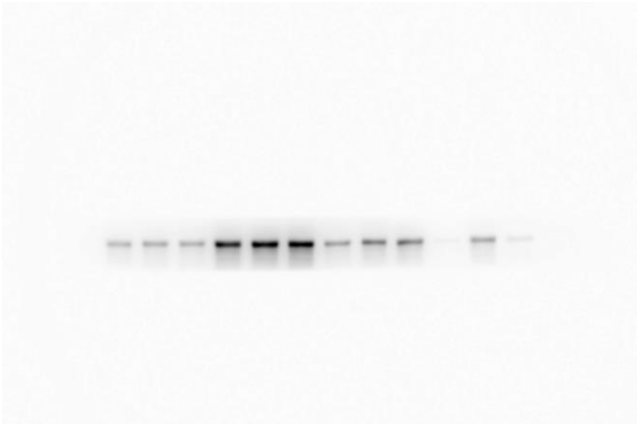

scWAT-HSP90

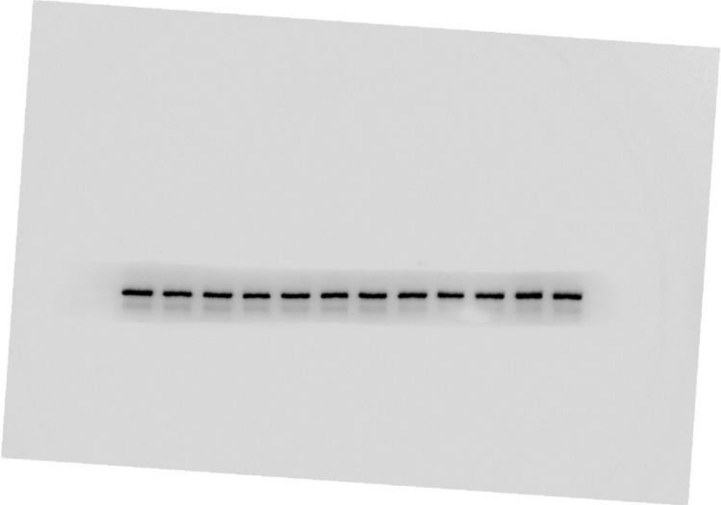

eWAT-HSP90

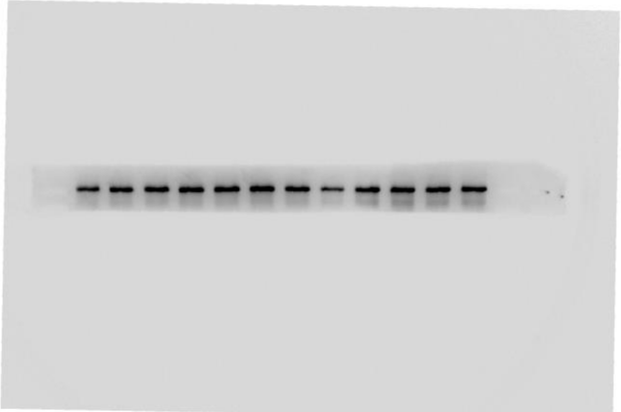

Fig5H

eWAT-pAKT

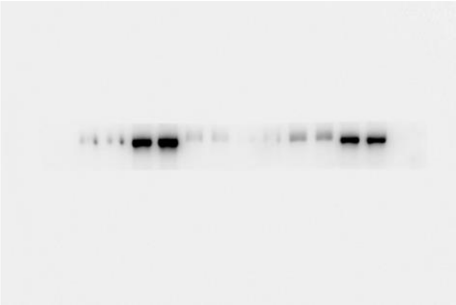

eWAT-HSP90

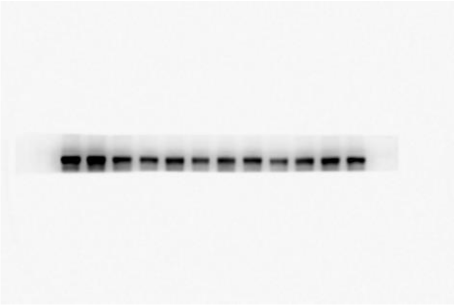

eWAT-AKT

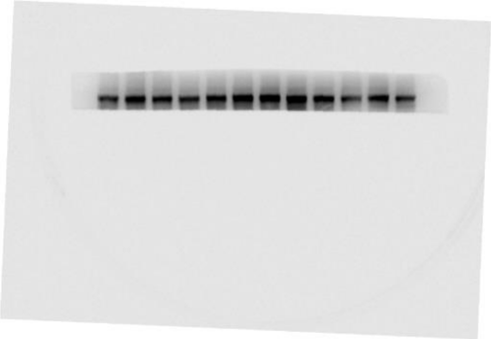

Liver-pAKT

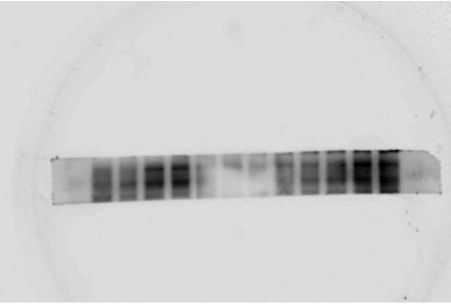

Liver-HSP90

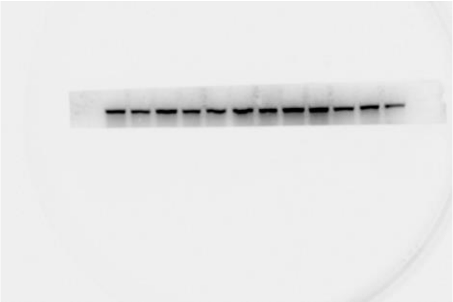

Liver-AKT

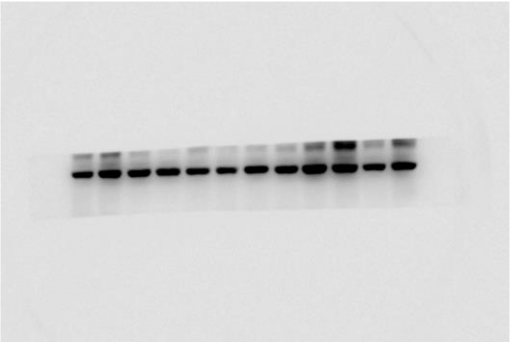

Muscle-pAKT

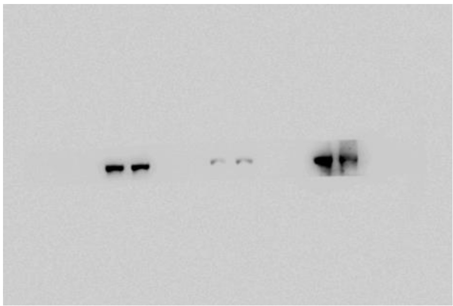

Muscle-HSP90

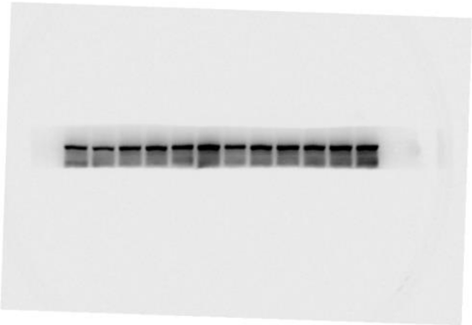

Muscle-AKT

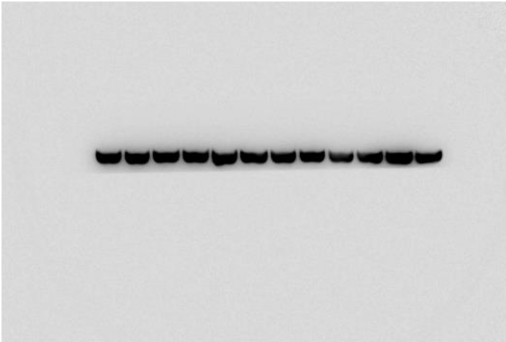

FigS1A

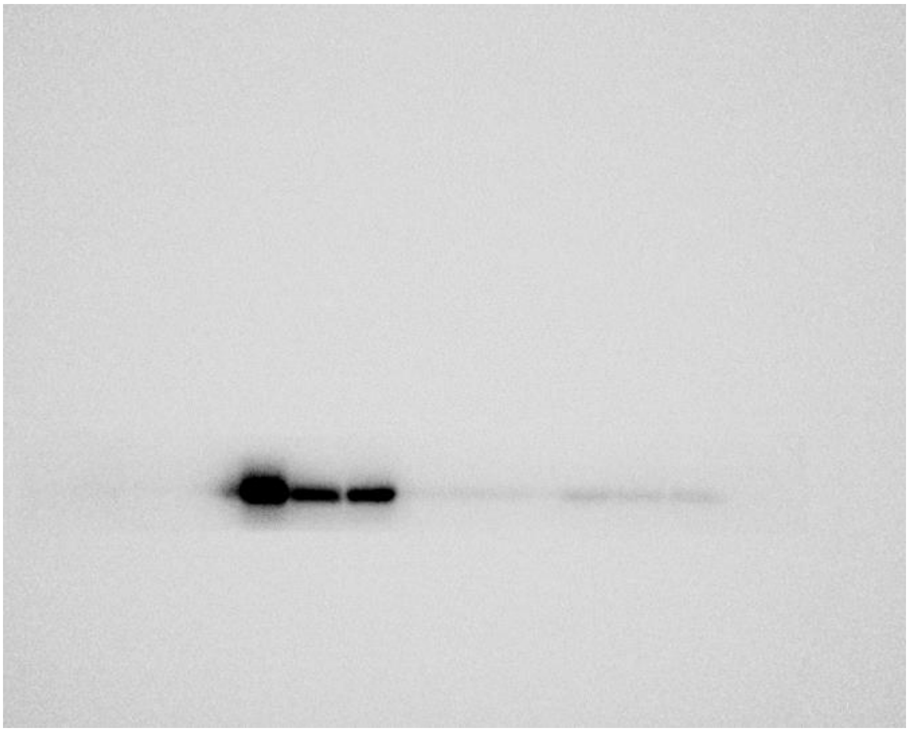

UCP1

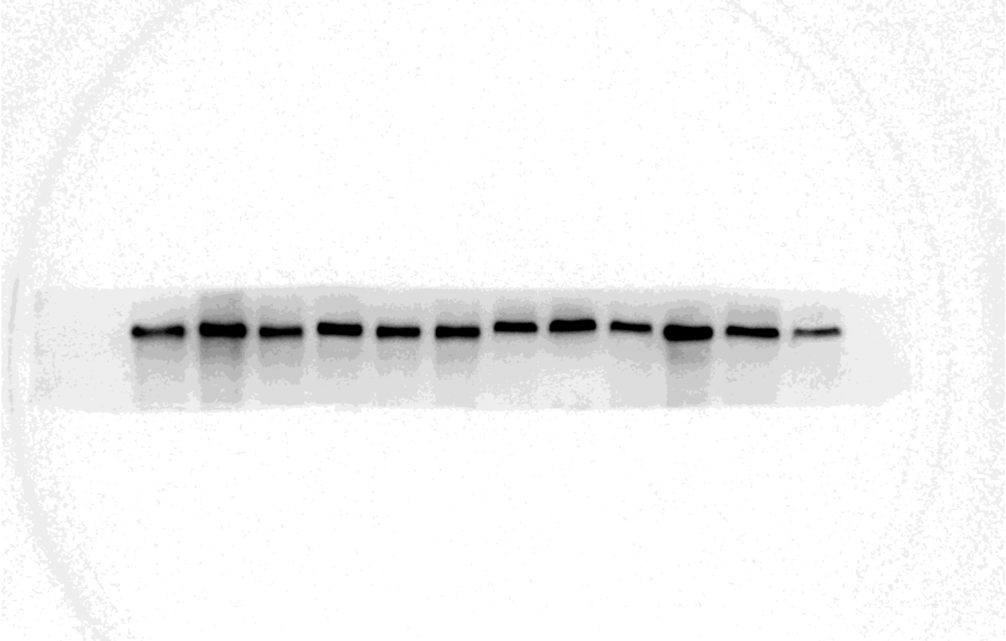

HSP90

FigS1D

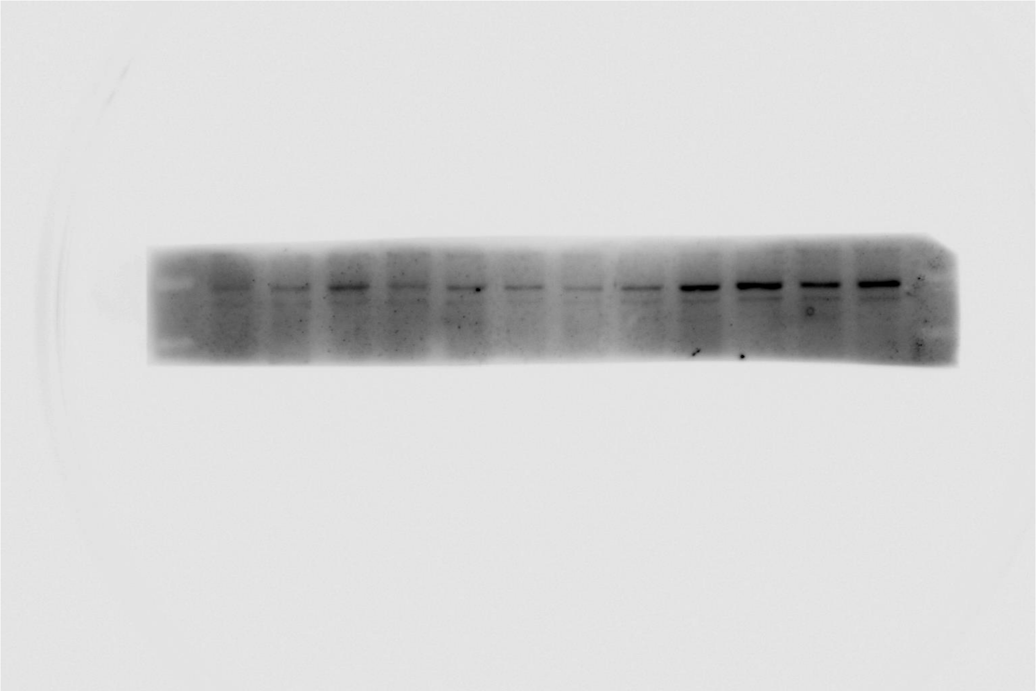

IL17RB

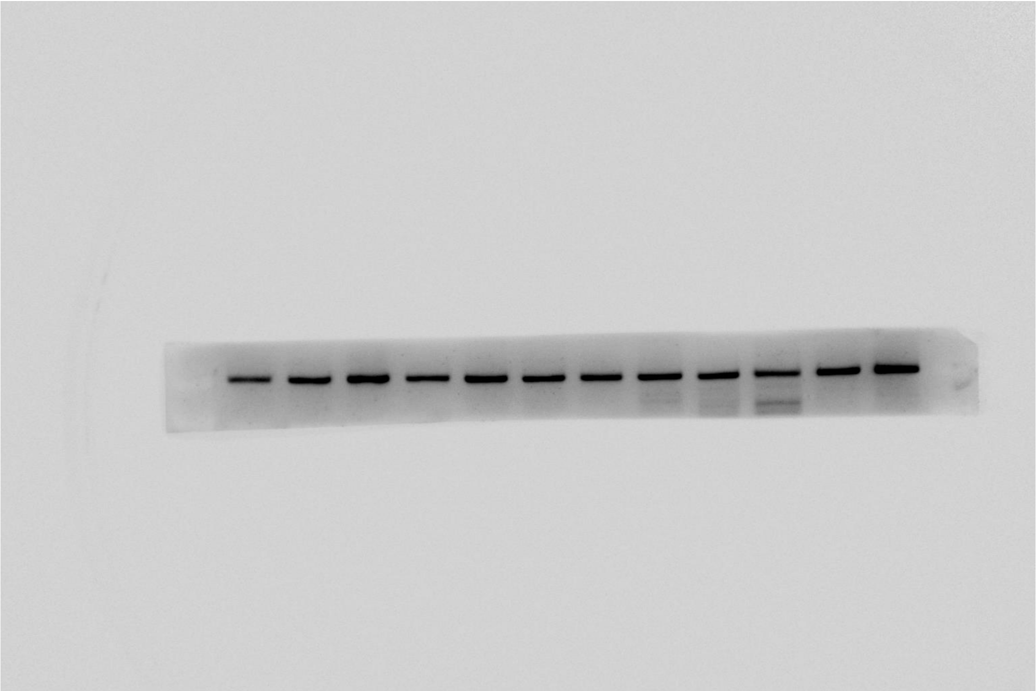

HSP90

FigS1G

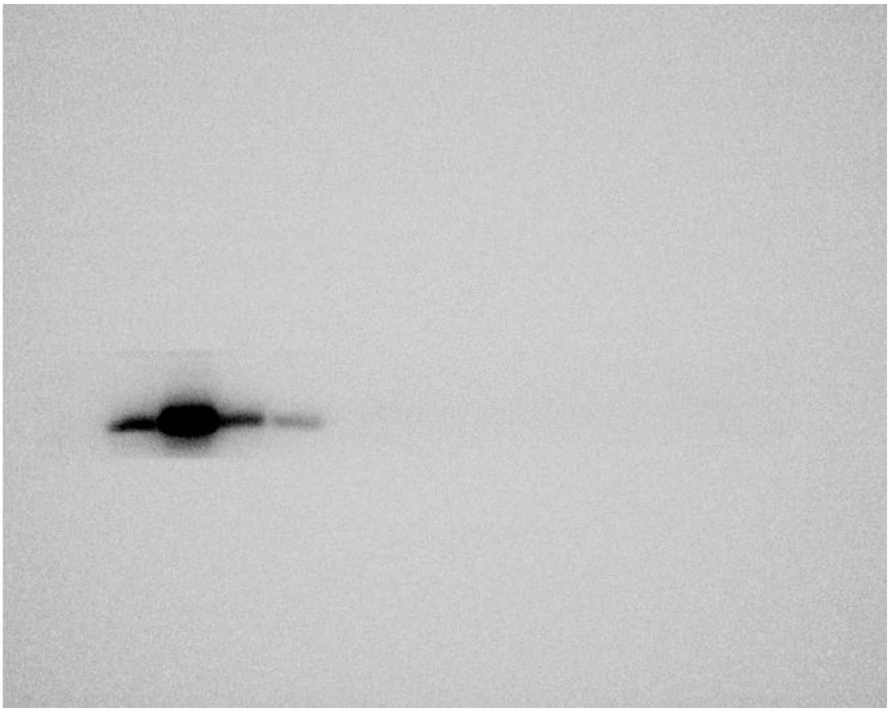

UCP1

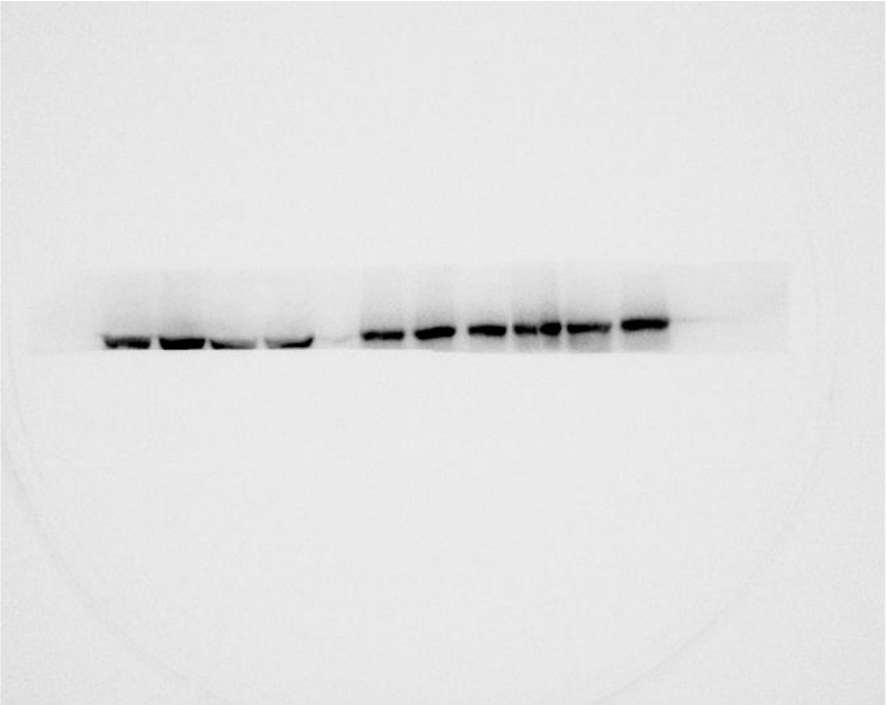

HSP90

FigS1J

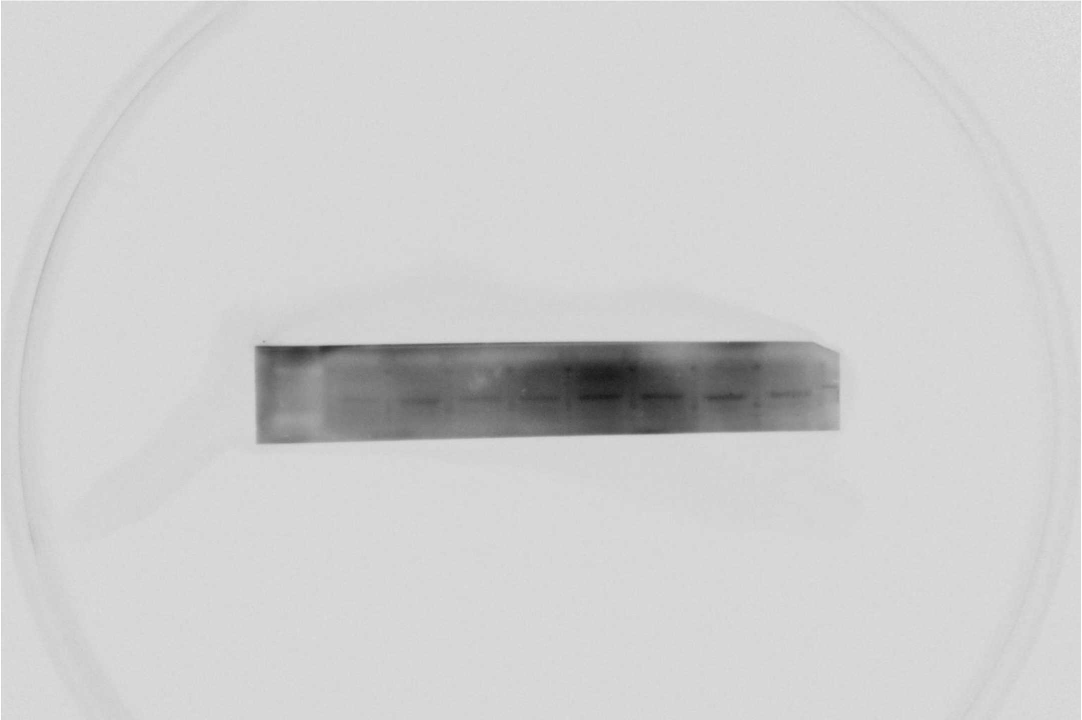

IL17RB

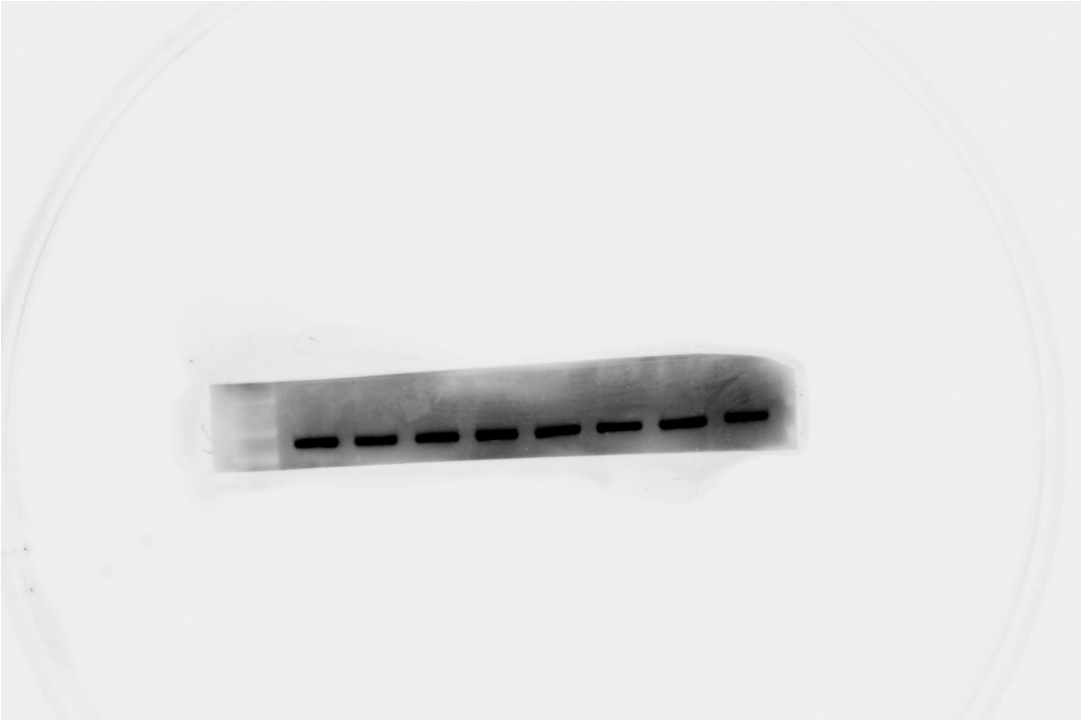

HSP90

FigS3A

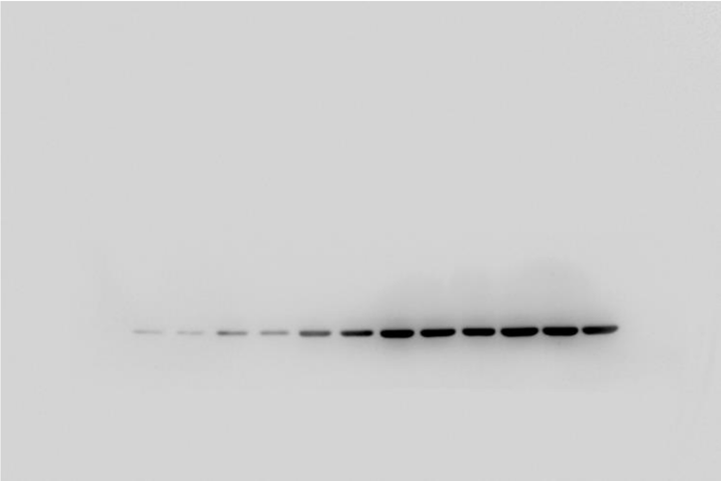

scWAT-UCP1

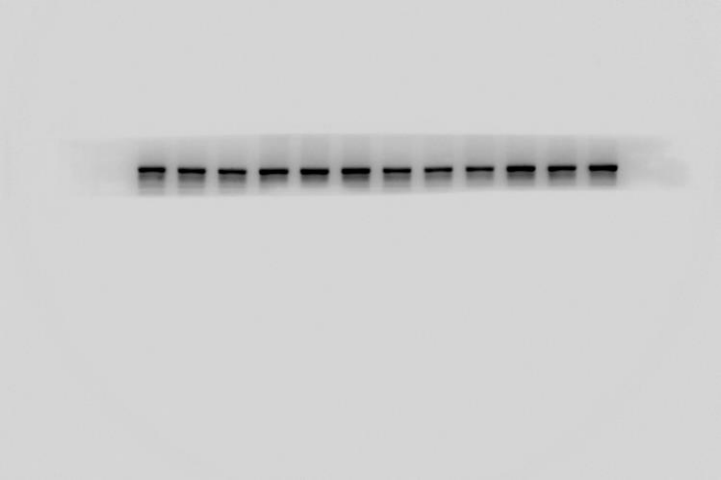

scWAT-HSP90

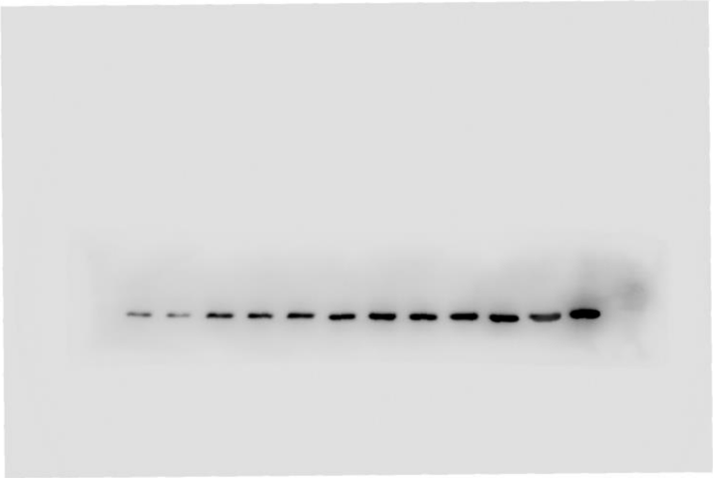

eWAT-UCP1

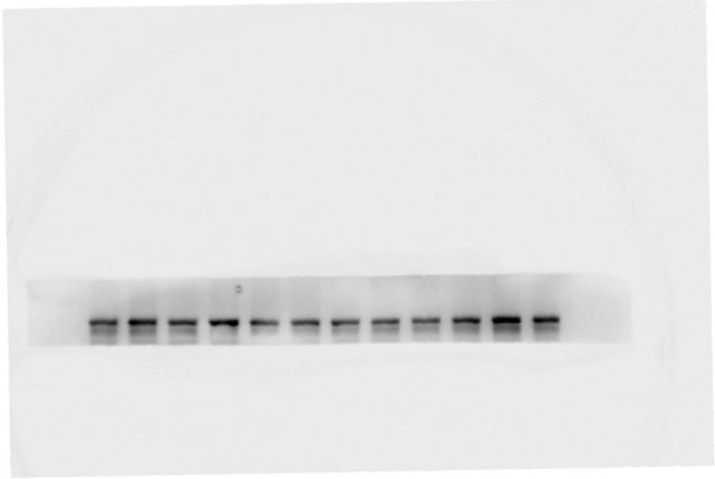

eWAT-HSP90
