# Supplementary material for: IL-25–induced shifts in macrophage polarization promote development of beige fat and improve metabolic homeostasis in mice
Source: PLoS Biol. 2021 Aug 5;19(8):e3001348. doi: 10.1371/journal.pbio.3001348 (PMC8341513; doi:10.1371/journal.pbio.3001348)
Supplement: Attachment — Submitted filename: point to point response [PBIOLOGY-D-18-01188R1].docx [file pbio.3001348.s006.docx]

Dear Dr Yang,

Thank you very much for submitting your manuscript entitled "IL-25 induces beige fat to improve metabolic homeostasis via macrophage and innervation" for review as a Research Article by PLOS Biology. As with all papers reviewed by the journal, yours was assessed and discussed by the PLOS Biology editors, by an academic editor with relevant expertise and by three independent reviewers.

Based on the reviews, I regret that we will not be able to accept this manuscript for publication in the journal. I hope that you will find the reviewers' feedback helpful as you consider how to proceed with this work.

The rationale behind this decision is that reviewers find that the study remains largely descriptive and that more mechanistic evidence is needed to draw more convincing conclusions. Given the comments from the reviewers, our Academic Editor advises, and we agree, that genetic deletion experiments, which are requested by reviewers 1 and 2, and the additional mechanistic insights suggested by reviewer 2, are essential for this study to meet our standard of publication. 

At this point, we can't know whether new data would support your conclusions, and as producing the data would require significant effort, we cannot in good faith invite resubmission. However, if you are willing to take on the extra work involved and comprehensively address the reviewers' concerns - and I must emphasize that this would be your choice - we would be willing to consider an extensively revised version as a new submission. As such, the article would receive a new number and submission date and we would evaluate it against any work published in the interim that might undermine the novelty. Although we would try our best to engage the same academic editor and reviewers, please be aware that this isn't always possible. Also, if the new experiments and data introduce new areas that require review we might need to consult additional/new reviewers. The new data would all need to be evaluated as if a first submission and further revisions might prove necessary.

We appreciate that the scale of the requested additional work is significant and you may well prefer to pursue publication of this work elsewhere. At this point, your manuscript is no longer under active consideration at PLOS Biology.

Please do feel free to contact me if you would like to discuss next steps for your manuscript. We might also be able to facilitate a faster consideration at one of the other PLOS journals; if this is of interest to you, please let me know.

If you do make the choice to revise and re-submit to PLOS Biology, you should provide a point-by-point response to the reviewers' comments when re-submitting. Please include this as a separate file, labelled 'Response to Reviewers'. Please also provide the tracking number of this manuscript [PBIOLOGY-D-18-01188R1] in the 'previous interaction' section of the online submission form and in your cover letter when re-submitting.

Sincerely,

Di Jiang
PLOS Biology

------------------------------------------------------------------------
Reviewer remarks:

Reviewer #1: Regulation on adipose tissue activity and adaptive remodeling is important for metabolic homeostasis. This study showed that b3-adrenergic receptor agonist stimulation or cold exposure led to upregulation of IL-25 and its receptor IL-17B in white adipose tissues. Moreover, administration of exogenous IL-25 could induce browning effect in the white adipose tissues. In addition, macrophages were proposed to be regulated by IL-25 in inducing the browning process. And UCP1 also functioned to mediate the improvement on glucose metabolism by IL-25 in diet-induced obese mice.  This study has revealed a potentially important role of IL-25 in regulating adipose tissue biology, thereby shifting metabolic balance under obese conditions.  

Major points: 
1. This study focused on the effects on browning by administration of exogenous IL-25. Evidences for its involvement under certain physiological settings are lacking. Loss of function study using blockade or genetic deletion targeting the ligand or receptor would provide stronger support for its important role in regulating adipose tissue biology. Since increases on both protein and mRNA levels of endogenous IL-25 and IL-17RB were shown here, studies to intervene its functions under physiological conditions would provide more insights.  
Answer:

Previously we reported that nematode infection stimulated type 2 immunity by releasing macrophage-responsive Th2 interleukins (ILs) including IL-25 (*to see the reference #15. Yang Z, Grinchuk V, Urban JF, Jr., Bohl J, Sun R, Notari L, et al. Macrophages as IL-25/IL-33-responsive cells play an important role in the induction of type 2 immunity. PLoS One. 2013;8(3):e59441.*) and parasitic worm infection fights obesity, related metabolic disorders (*To see the reference #16. Z. Yang, et al. Parasitic Nematode-Induced Modulation of Body Weight and Associated Metabolic Dysfunction in Mouse Models of Obesity. Infection and Immunity, 2013, 81(6): 1905–1914*). We also know that IL-25 is a mucosal immunity-derived cytokines stimulated by nematode infection and is involved in lipid metabolism (*To see the reference #19. J. Feng, et al. IL-25 stimulates M2 macrophage polarization and thereby promotes mitochondrial respiratory capacity and lipolysis in adipose tissues against obesity. Cell Mol Immunol. 2018 May;15(5):493-505*). This study focused on the effects on browning by administration of exogenous IL-25 to clarify the mechanism. So, We added the background evidence for IL-25’s involvement under the nematode infection and metabolic disorder physiological setting in the introduction of resubmitted manuscript. We constructed IL-25 knockout mice using Crisper cas9 technology, and used it as a model to carry out relevant functional experiments. We found that after knocking out IL-25, the body weight of mice and their fasting blood glucose increased and insulin tolerance test (ITT) and glucose tolerance test (GTT) deteriorated and the fat droplets of brown and white fat increased, respectively (*To see Figure 6. Depletion of IL-25 deteriorate Glycolipid and energy metabolism*).

2. The cellular expression of IL-17RB in the white adipose tissues was shown in Fig 1D. Co-staining of IL-17RB and PLIN1 would clarify whether IL-17RB was expressed in adipocytes. The cell morphology suggested that IL-17RB could be expressed predominantly in adipocytes. While on the other hand, no effect of IL-25 on 3T3-L1 was observed in vitro. Those data should be reconciled to explain how IL-25 may function on its target cell types while being excluded from its direct effect on adipocytes. 
Answer:

We Co-stained IL-17RB with PLIN1 (the marker of adipocyte) and CD68 (the marker of macrophage) in adipose tissue by immunofluorescence（To see Figure 1J）. It was found that the expression of IL-7RB in adipose tissue was lower at normal temperature, increased significantly at cold stimulation, and mainly expressed in macrophages, but less in adipocytes.

3. Ucp1 deficient mice were shown before to be resistant to diet-induced obesity when housed at 20°C (Liu et al. JCI, 2003). Comparable fasting glucose was observed in the knockout vs wildtype mice in the above mentioned study. However, Ucp1 knockout in the current report showed compromised insulin and glucose sensitivity when housed at 21°C in this study (Fig S3 and Fig 6). The inconsistent results should be clarified here.

Answer:

Liu et al. reported that Ucp1 deficient mice were resistant to diet-induced obesity when housed at 20°C, while this resistance was quickly reversed when the ambient temperature was increased to 27°C (Liu et al. Paradoxical resistance to diet-induced obesity in UCP1-deficient mice. J. Clin. Invest. 2003, 111:399–407). Although shivering under cold is one alternative mechanism of thermogenesis that is probably used in UCP1-deficient mice, others including nonshivering thermogenesis such as thyroid function were also involved. By the 17th week, the differences in body weights between the two groups of WT and UCP1-KO mice were statistically insignificant. Our current study also showed there was no statistically significant differences in body weights between WT and UCP1-KO mice when factually housed at 24°C closed to the above reported 27°C though the temperature were set at 21°C, which was consistent to the above article。When animals are exposed to cold (Usually, the temperature of cold stimulation is 4-16 °C), the expression of UCP1 gene is increased to activate thermogenesis, while, in the regular animal facility, the temperature is usually 22°C, in this study, is 24°C, therefore, there is little difference of UCP1 between WT and KO mice, as well as little difference of body weight.

4. Macrophage depletion alone using clodronate showed similar effects on improvement of glucose metabolism compared to IL-25 treatment alone on HFD mice.  Those data raised questions as whether macrophages and IL-25 function independently to regulate glucose metabolism by large. Also, as discussed in the current study, the involvement of macrophages and IL-4-IL-4Ra signal in the adaptive thermogenesis was challenged in the previous report (Fischer et al. Nature Medicine, 2017), and how macrophages and IL-4 signal were involved in browning mediated by IL-25 in the current study is suggested to be better clarified. 
Answer:

Our data approved the conclusion that IL-25 regulate glucose metabolism partially dependent on macrophage, similar to the reviewer’s opinion that macrophage and IL-25 function independently to regulate glucose metabolism by large. IL-25 induces the beige fat not only by promoting macrophage to secrete IL-4, but also by promoting innervation to secrete catecholamines. Therefore, when macrophage was depleted by clodronate liposome, IL-25 still could induce the beige fat.

The previous report (Fischer et al. Nature Medicine, 2017) just identified that alternatively-activated macrophages did not synthesize catecholamines to contribute to adipose tissue adaptive thermogenesis, but not to indicate neither M2 macrophage nor catecholamines could induce the beige fat. Some previous publications reported that M2 macrophage or IL-4 could induce beige fat. Our current data just identify that IL-25 induce the innervation to secrete catecholamines, but not by polarizing macrophage to secrete catecholamines.

5. The differential regulation on body weight and glucose metabolism by IL-25 should be further clarified. The current study showed that in the macrophage-depleted or Ucp1 deficient mice (Fig S4 and Fig6), administration of exogenous IL-25 led to decreased body weight, fat mass and fat accumulation in liver, which in most cases result in improvement in glucose sensitivity and insulin sensitivity but failed doing so here. 
Answer:

This article focuses on the lipid metabolism and its associated aspects such as body weight, fat mass, fat accumulation in liver, thermogenesis by IL-25 on the view of beige fat transition, not glucose metabolism, though the current data including some glucose metabolism such as GTT, ITT. We mentioned the glucose metabolism just for the consideration of metabolic homeostasis. It is another story to clarify the detailed mechanism that IL-25 affects on the glucose metabolism. In fact we are going to clarify the mechanism of IL-25 on glucose metabolism in a new item.

Minor points:
1. Fig 1 showed the increase of IL-17RB on protein and mRNA levels using western-blot (1A), immunohistochemistry (1C) and ELISA (1E). While the protein level detected by immunohistochemistry showed much larger increase.  The discrepancy on the results using various methods should be clarified.
Answer:

We carefully re-checked these results listed by the reviewer and found that IL-17RB was examined by western-blot (1A) and immunohistochemistry (1C), but not by ELISA (1E) (In fact it is IL-25, not IL-17RB). In this manuscript, we try our best to make great afford and avoid the discrepancy on the results using various methods. Here, we thank the reviewer’s remindness.

2. The immunohistrochemistry on TH in Fig 4 rather showed cellular morphology instead of axons as claimed in the study.
Answer:

Thanks yours reminder that the immunohistrochemistry on TH in Fig 4 rather showed cellular morphology instead of axons as claimed in the study. In the Figure 4 in the 1^st^ submitted manuscript, We have change the description of “Histological staining of TH in scWAT and eWAT showed more sympathetic axons” into “Histological staining of TH and Neurofilament (NF) in scWAT and eWAT showed more transverse section of sympathetic axons”. We identified the innervation by examining both tyrosine hydroxylase (TH), namely the enzyme for synthesizing dopa (the precursor of neuron transmitter epinephorine), and neuron marker Neurofilament (NF).

Reviewer #2: This manuscript reports the investigation of the effects of IL-25 on white adipose tissue (WAT) browning and metabolic homeostasis in mouse models. The presented results showed increased IL-25 signaling in WAT upon β3-adrenergic agonist stimulation or cold exposure, and the data indicated that IL-25 might increase the levels of IL-4 and IL-13 to stimulate the browning of adipose tissue via inducing macrophage alternative activation, thereby improving the metabolic homeostasis in DIO mice.

Overall, the manuscript described some interesting observations, suggesting a potential connection between IL-25 signaling and WAT browning. However, the current study remains largely descriptive and more mechanistic evidence is needed to draw a more convincing conclusion. To strengthen the significance and mechanistic understanding of the metabolic actions of IL-25, the IL-25 knockout model should be considered and several important issues also need to be addressed.

Specific points:
1. One major issue is that no substantial evidence is provided to show that there are increases in innervated sympathetic axons in the WAT of IL-25-treated mice. In addition to an increased TH staining, as shown in Figure 4C, a co-staining of TH with nerve fiber marker would be more informative. It would also be interesting to examine the possible interaction between the innervation and alternatively activated macrophages in WAT of IL-25-treated mice, like that reported in BAT by Dr. Steffen Jung’s group recently (Nat Immunol. 2017).

Answer:

To further identify that there are increases in innervated sympathetic axon in the WAT of IL-25-treated mice, besides tyrosine hydroxylase (TH), we further examined the neuron marker Neurofilament (NF), which were co-stained with tyrosine hydroxylase (TH, the enzyme for synthesizing dopa, the precursor of neuron transmitter epinephrine) and shown in the figure 4.

It is really interesting that Dr. Steffen Jung’s group removed the skin above interscapular region to make tissue denervation and found significantly decrease the expression of TH and UCP1, thus finally to examine the possible interaction between the innervation and macrophage. Similarly, We applied clodronate-liposome to deplete macrophage or UCP^-/-^ mice to examine the interaction between macrophage and innervation from the aspect of macrophage and UCP1. We found that IL-25 induces the beige fat to improve metabolic homeostasis partially dependent on the macrophage and UCP1. In order to comprehend the relationship between tissue resident macrophage and innervation, we also cited above reference as #38 in the resubmitted manuscript.

2. The authors concluded that IL-25 could stimulate IL-4/IL-13 release in scWAT and eWAT to promote macrophage alternative activation and browning, largely based on the results shown in Fig 3B and 3C. However, in addition to the detection of increased Arg-1 and Ym-1 mRNA expression, it is necessary to perform flow cytometry analysis to examine the polarized activation states of macrophages. Given that the cell sources of IL-4/IL-13 production are likely to be eosinophils, it is desirable to investigate how IL-25 may regulate eosinophils to produce and release IL-4/IL-13.

Answer:

We have examined two different original isolated macrophages using flow cytometry (FCM) stained with F4/80 (marker of general macrophage) and IL-13: one is from adipose tissue residental macrophages (ATRM), the other is from bone-marrow-derived macrophages (BMDM). We succeeded to examine BMDM with FCM and found that IL-25 increase F4/80-IL13 double staining macrophage from 0.78 to 2.42, which increased nearly 3-folds (To see the following figures). But we failed to do the experiment based on ATRM.

3. It would be highly valuable to look into the cellular sources in adipose tissues for producing IL-25 upon cold exposure or β3-adrenergic agonist stimulation.

Answer:

To look into the cellular sources in adipose tissues for producing IL-25, we not only examined the co-localization of IL-25 and perilipin (the protein located around lipid droplet and serving as a marker of adipocyte) with immunofluorescent double-staining of anti-IL25/anti-Perilipin antibody in adipose tissue of mice upon cold exposure, but also examined the expression of IL-25 in 3T3-L1 adipocyte upon β3-adrenergic agonist stimulation and in macrophages upon IL-4 stimulation, respectively. We found that both adipocytes and macrophages can produce IL-25, and significantly increase upon cold exposure or β3-adrenergic agonist stimulation (To see the resubmitted Figure 1K).

4. For the author’s claim that IL-25 can link macrophage activation to thermogenesis, analysis of energy expenditure by CLAMS and the core body temperature should be performed under cold exposure.

Answer:

To analyze the energy expenditure We performed the experiment of CLAMS (Comprehensive Lab Animal Monitoring System, CLAMS) based on the IL-25KO and WT models, and found the deletion of IL-25 leads to lower consumption of O_2_, CO_2_ and calorie, higher respiration quotient, lower temperature, which meant that IL-25 can affect thermogenesis without changing the food intake (To see the resubmitted Figure 6 and S2B and S2C Fig).

5. Usually, it is very hard to detect UCP1 protein expression by immunoblotting in eWAT of mice in the B6 background, especially when fed a HFD. The WAT UCP1 protein levels from the presented data appeared to be highly variable from different experiments. Samples of scWAT and eWAT should be analyzed in the same gel to examine the relative expression level of UCP1, and UCP1 KO control samples need to be used.

Answer:

We loaded the samples of scWAT and eWAT in the same gel to examine the relative expression level of UCP1 and were auto-exposured to snap, which were shown in the resubmitted Figure S1A and S1G. We found that the UCP1 signal of eWAT was much weaker than that of scWAT. If we loaded the samples of scWAT and eWAT in the different gels and auto-exposure to snap, eWAT group obviously cost much longer time to get similar intensity band.

All UCP1KO mice were examine to be identified as homozygous knockout allele and the PCR band were shown in the following picture. Therefore, we considered there were no UCP1 protein expression in the adipose tissue of UCP1KO mice.

6. In Figure 6, the significance marks for the statistical analysis are not clear and even confusing.

Answer:

We did the proofread and made the statistical analysis more clear.

7. The working model needs to be improved for clarity and relevance. Professional language editing would be helpful to make the paper more reader-friendly.
Answer:

We modified the working model and improved the clarity and scientific relevance. We also proofread the professional language editing to make the paper more reader-friendly.

Reviewer #3: Authors examined the effects of IL-25 signaling in adipose thermogenesis and fat-induced metabolic disorders. It is interesting to see that IL-25 induces WAT browning and white fat innervation. Overall, the data presented seem to support this main conclusion. Evidence in support of the role of alternatively activated macrophages in mediating IL-25 effects on WAT browning is relatively weak. Specific comments are:

1. Figure 1D: UCP1-positive cells do not seem to be those strongly positive for IL-25. Any explanations?
Answer:

Because both adipocytes and macrophages can produce IL-25, and IL-25 can significantly increase upon cold exposure or β3-adrenergic agonist stimulation, while UCP1 only increases in the adipocyte but not in macrophages, therefore, some UCP1-positive cells do not seem to be those strongly positive for IL-25.

2. A 2-day cold exposure may be too short to induce IL-17RB protein expression. Why did the authors do long (7 day) cold exposure? Also, why did the author treat the mice with CL for only 2 days. The treatment duration is not the standard one.
Answer:

We treated the mice upon cold exposure or β3-adrenergic agonist stimulation in a time-course manner and found that both IL-17RB and UCP1 protein became up-regulated. Therefore, some experiments were performed only for 2 days, some for 5 days for the reasons of samples collection. Afterall, the phenomenon of beige fat induction became obviously.

3. The normal value for serum IL-25 is about 40 pg/ml (Fig. 1J). What was the concentration of IL-25 post injection of 1000 ng IL-25? 
Answer:

We examined the concentration of IL-25 post i.p. injection of 1000ng/mouse IL-25 for 2 weeks in an every-other-day manner and the concentration of IL-25 is from 84.2 to 282 pg/ml.

4. Fig. 3A: It would be helpful to include more pro-inflammatory genes and to analyze adipose macrophages using FACS sorting for this purpose.
Answer:

We have tried to examine the expression of interleukin such as IL-13 using FACS and found that IL-25 increase IL13 expression in macrophages from 0.78 to 2.42, which increased nearly 3-folds (To see the above figure).

5. Fig. 5E: It would help this reviewer if the total body weight were shown at each time point. This would help explain the GTT results (Fig. 5G). In general, when mice are leaner, they are glucose tolerant and insulin sensitive.
Answer:

We Just examined the body weight during the treatment of IL-25 and the results were shown in the following figure.

6. Fig. 5F and other figures showed the blood glucose concentrations. What about blood insulin levels at the same time? This piece of data would help assess systemic insulin sensitivity. 
Answer:

We examined the blood insulin level at the end point of IL-25-treated mice and shown that IL-25 significantly increases the blood insulin level. We also examined the blood insulin level of IL-25KO mice and shown it is decreased. The results were listed in the following figures.

7. Why did the IL-25-injected mice gain less weight? Any changes in daily food intake, physical activities and total body energy expenditure? 
Answer:

In our previous reports, We identified that IL-25 can promote lipolysis by up-regulating lipolytic enzymes, and here we further identified that IL-25 can induce the beige fat to enhance thermogenesis. The two mechanism might be the reasons that IL-25-injected mice gain less weight. During treatment, there was no obvious changes in daily food intake, physical activities, but there was some changes in energy expenditure.

We also applied the IL-25KO and WT models to perform the experiment of CLAMS (Comprehensive Lab Animal Monitoring System, CLAMS) and observe the metabolic effects on mice, and found the deletion of IL-25 leads to lower consumption of O_2_, CO_2_ and calorie, higher respiration quotient, lower temperature, which meant that IL-25 can affect thermogenesis without changing the food intake (To see the resubmitted Figure 6 and S2B and S2C Fig).

8. Did IL-25 suppress satiety? Was the diet-induced thermogenesis inhibited? If yes, some of the phenotypes may be central, not peripheral. 
Answer:

We didn’t observe the above phenomenon.

9. Fig. S2G: It is better to normalize liver weight by total body weight.
Answer:

We have normalized liver mass by total body weight which were shown in the manuscript as listed in the following figure.

10. Numerous grammatical errors throughout the manuscript.
Answer:

We proofread the professional language editing to make the paper more reader-friendly.
