# Supplementary material for: IL-25–induced shifts in macrophage polarization promote development of beige fat and improve metabolic homeostasis in mice
Source: PLoS Biol. 2021 Aug 5;19(8):e3001348. doi: 10.1371/journal.pbio.3001348 (PMC8341513; doi:10.1371/journal.pbio.3001348)
Supplement: Attachment — Submitted filename: point to point response-[PBIOLOGY-D-20-02642R2].docx [file pbio.3001348.s008.docx]

**Reviewer remarks：**

**Reviewer #1**: The authors conducted a substantial amount of additional experiments and addressed most, if not all, of the questions and concerns that were raised. The revised manuscript has been significantly improved for publication with more careful language editing.

Answer：Thanks.

**Reviewer #2**: The authors have not satisfactorily addressed some major questions. For examples, The IL-25-injected animals were more glucose tolerant and insulin sensitive. You would expect to see reduced blood insulin levels. Unfortunately, the opposite was observed according to the rebuttal, which raises a serious question about the beneficial role of IL-25 in metabolic disorders. It is highly possible that IL-25-injected animals had some issues in the gastrointestinal organ causing less weight gain, which affected some metabolic parameters. IL-25 KO mice only showed modest changes in weight gain and ITT. Also it is unclear why the high fat-fed control mice weighed less than 25 grams. The beiging effect (Fig. 6B) is quite unusual because 48h cold exposure cannot induce that much beiging morphologically.

**Point 1：**The IL-25-injected animals were more glucose tolerant and insulin sensitive. You would expect to see reduced blood insulin levels. Unfortunately, the opposite was observed according to the rebuttal, which raises a serious question about the beneficial role of IL-25 in metabolic disorders. It is highly possible that IL-25-injected animals had some issues in the gastrointestinal organ causing less weight gain, which affected some metabolic parameters.

Answer：

FigS2 G showed no difference in fasting plasma insulin levels between wild-type mice and IL25 knockout mice. As for the conflicting results observed, we attribute them to an error caused by insufficient fasting time (less than 4 hours) which is why we did not add the two figures in the manuscript. Therefore, we conducted another animal experiment to detect changes in plasma insulin and glucose levels after IL25 treatment in High-fat diet mice after 8 hours fasting time. The results were listed in the Figure 5E, as well as the following.

**Point 2**：IL-25 KO mice only showed modest changes in weight gain. Also it is unclear why the high fat-fed control mice weighed less than 25 grams.

Answer：

The previous result showed the body weight of WT and IL-25 knockout mice after only 4 weeks on a normal diet or a high-fat diet, which is why the high fat-fed control mice weighed less than 25 grams. We revised the result of body weight after 12 weeks on a high-fat diet in the Fig 6A as well as the following.

**Point 3**：IL-25 KO mice only showed modest changes in ITT.

Answer：

The reason that ITT of IL-25 knockout mice showed only moderate changes was that FIG6 D-E showed GTT and ITT of WT and IL-25 KO mice after only 4 weeks on a high-fat diet. We revised the results after 12 weeks on a high-fat diet in the Fig 6D-E as well as the following.

**Point 4**：The beiging effect (Fig. 6B) is quite unusual because 48h cold exposure cannot induce that much beiging morphologically.

Answer:

We chose the image with the strongest signal previously, but now we choose the more representative and more average image in the Fig 6B as well as the following.

**Reviewer #3**: In the current study, Li et al. reported that IL-25 could regulate the white adipose tissue (WAT) browning process. IL25 and IL-17RB protein levels increased by the stimulation of b3-adrenergic receptor agonist or cold exposure. Administration of recombinant IL-25 could induce white adipose tissue browning and alternatively activated macrophages were proposed to be IL-25 responsive cells during this process. The authors further showed IL-25 treatment improved glucose metabolism in the high-fat diet-fed animals, and IL-25 knockout mice were protected from DIO. Overall, the data presented here indicate an interesting role that IL-25 plays to regulate WAT browning.

Specific comments:

1. The description should be more accurate and inclusive in the introduction. For examples, "although the thermogenic tissue is almost lacking in adults" should refer to human and adaptive thermogenesis of classical brown adipose tissues, and regarding the lineage of beige adipocytes, the Myf5 positive cells have been shown to contribute partly to brite/beige cells in the scWAT. The discussion should be more coherent and focused.

Answer：

Thanks for reviewer’s advice and we improve the statement in the manuscript. For example as the following:

In the first paragraph of introduction:

Two types of thermogenic adipocytes including as classical brown adipocytes (developmental origin from Myf5^+^ cells) and beige (also referred to as brite) adipocytes (origin from Myf5^-^ cells) both with uncoupling protein 1 (UCP1) in humans, control energy and glucose homeostasis. Activation of UCP1-positive adipocytes would release heat via uncoupled oxidation respiratory chain for ATP synthesis [2]. Adaptive thermogenic adipocytes, especially regarding the lineage of beige adipocytes composed of heterogeneous cell populations in multiple white adipose tissue (WAT) depots, could exhibit UCP1-dependent thermogenic capacity when stimulated by certain external cues, such as chronic cold exposure, exercise, and long-term treatment with PPARγ agonist (e.g. rosiglitazone), although human classical brown adipocytes in the interscapular region gradually disappear with age. While in adults, ^18^F-FDG-PET/CT scans have identified active brown adipose tissue (BAT) depots in the cervical, supraclavicular, axillary, and paravertebral regions. Therefore, besides the classical brown fat, the other thermogenic tissue named beige fat tissue could also be a potential target for treatment of obesity.

In the discussion:

We delete the following sentence：

Using IL-25 to pharmacologically activate alternatively activated macrophages, we were able to place alternatively activated macrophages to the environment of IL-25-induced browning WAT.

In the fifth paragraph of discussion, We delete the following sentence：

After the effect of IL-25-inducing beige fat had been investigated, the role of which on the thermogenesis and metabolic homeostasis was further explored. In this model, IL-25 decreased body weight gain, improved glucose disposal and insulin sensitivity.

IL-25 failed to improve glucose tolerance and insulin sensitivity in DIO mice with macrophages depletion. The anti-obesity effect of IL-25 did not require macrophages for that depletion of macrophages could not impaired IL-25-mediated lowering of body weight.

2. The role of Ucp1 vs beige fats was not clearly explained. The improvement of glucose metabolism in DIO by IL-25 administration was dependent on Ucp1 and macrophages as in Fig7. But the body weight change and the fat pad decrease induced by IL-25 seems largely independent of Ucp1 or macrophages as in FigS3.

Answer：

The effect of IL-25 on the body weight change and the fat pad decrease IL-25 is not completely dependent on macrophages and beige fat, which may be related to the presence of other IL-25-responsive cells in the body, such as ILC-2 cells and eosinophils (Mitchell and O'Byrne, 2017).The activation of ILC-2 cells by IL-33 also induces the biogenesis of beige fat (Brestoff et al., 2015; Lee et al., 2015), and IL-25 can also promote lipolysis by directly upregulating the expression of lipase (Feng et al., 2018). In addition, UCP1 is one of the signature molecules of beige fat (others include Cox8b, Cidea et al). The beige fat is characterized as increased number of mitochondria, high expression of UCP1 and multilocular lipid droplets. Gene ablation of UCP1 partially inhibited the biogenesis of beige fat. Therefore, the effect of IL-25 on the body weight change and the fat pad decrease is not completely dependent on macrophages and UCP1.

3. The adipose tissues normally show high auto-fluorescent signals. To rule out the contribution of autofluorescence from adipocytes in immunostaining of IL-25, IL-17RB, and CD68, high-resolution images, and negative controls should be included. And isolated adipocytes and adipose macrophages using FACS sorting under cold challenge or CL stimulation would provide both gene expression and western blot data to consolidate the conclusion from immunostaining.

Answer：

We improved the results of Fig1 I-J as follows according the advice of reviewer. Fig I just shows the co-localization of IL-25, perilipin A and CD68 at normal temperature.

At the same time, we isolated primary mature adipocytes from adipose, and induced primary macrophages from monocyte isolated from spleen to provide the gene expression data to consolidate the conclusion from immunostaining as follows.

4. The conclusion of IL-25 on the regulation on TH innervation is not founded by the current dataset. It could be a correlation between browning and TH innervation change.

TH staining in Fig. 4D and 4F showed different morphology. The discrepancy in the results needs to be clarified.

Answer：

We drew the conclusion of IL-25 regulates the innervation of white adipose tissue by macrophage on the basis of Fig4 A-E (which showed that significantly more sympathetic axons in adipose tissue of IL-25-treated mice), Fig4 F (which presented that the effect of cold exposure on sympathetic distribution was blocked in IL-25^-/-^ mice) and Fig4 G (which showed that depletion of macrophages in DIO mice clearly blunted the IL-25 induced expression of TH and UCP1). In Figure4, the conclusion that IL25 regulates sympathetic nerve distribution was positively and negatively verified from the protein expression level (Figure 4A-B, G), mRNA expression level (Figure 4C), catecholamine expression level (Figure 4E) and morphology (Figure 4D, F), respectively. In summary, there may be a mutual promotion correlation between browning and TH innervation change, but IL-25 can induce the innervation of adipose tissue indeed and IL-25 knockout can block the effect of cold exposure on sympathetic distribution, indicating the role of IL-25 on the regulation on TH innervation. Therefore，we think the conclusion of IL-25 on the regulation on TH innervation is fully supported by the current dataset.

The reason of the different morphology in the previous results may be due to multilocular lipid droplets induced by cold exposure and different DAPI staining intensities. We improved the TH staining result in Fig 4F as follows.

5. Lack of scale bar in Fig. 1I; 1J; Fig. 4F; Fig. 6B.

Answer：The Scale Bar has been added.

6. Typo: mRNA expression of scWAT (arbitrary units) from Fig. 2B to Fig. S3B.

Answer：The typo has been corrected.

7. S1F, the figure should show how the data were normalized.

Answer：

The left and right vertical axis shows how the data were normalized. Adipose tissue (100 mg) was rinsed and homogenized in 1 ml of PBS. The homogenates were centrifuged for 5 min at 10,000 rpm for 10 min at 4 ℃ and then the supernate was assayed immediately. All samples were normalized to total tissue protein concentration.

8. The description of UCP1 expression levels in eWAT and western blots in various figures was not consistent.

Answer：

The expression level of UCP1 in scWAT is different from that in eWAT. Under the same exposure condition, eWAT signal is weaker than scWAT. Under the separate exposure condition, eWAT exposure time is longer than scWAT. Therefore, the results of simultaneous exposure (FigS1 A, G) are inconsistent with the results under the separate exposure condition (Fig2 A, D).
