# Supplementary material for: IL-25–induced shifts in macrophage polarization promote development of beige fat and improve metabolic homeostasis in mice
Source: PLoS Biol. 2021 Aug 5;19(8):e3001348. doi: 10.1371/journal.pbio.3001348 (PMC8341513; doi:10.1371/journal.pbio.3001348)
Supplement: Attachment — Submitted filename: response to reviewer remarks-[PBIOLOGY-D-20-02642R2]4th.docx [file pbio.3001348.s009.docx]

**Reviewer remarks：**

**Reviewer #2**: Authors appropriately addressed my previous comments.

Answer：Thanks.

**Reviewer #3**: The questions are addressed. Some details: for example, some words within the figures are distorted.

Answer：

Thanks for reviewer’s advice. We checked all the figures again and revised some possible distorted figures as the following:

Fig3 B: IL-1β is corrected as Il-1β in order to consistent with others.

Fig4 B, F and G: stretch out and draw back the figures as a fixed magnification ratio.

Fig5 D is deleted the background word in the X-axis.

Fig5 E, F: stretch out and draw back the figures as a fixed magnification ratio.

Fig6 F, G, I: The unit of Y-axis is corrected from ml·g^-1^·h^-1^ into ml·g^-1^·min^-1^.

Fig6 I: The Y-axis is corrected from Kcal(g^-1^) into Energy expenditure (Kcal·g^-1^·h^-1^).

Fig7 B: The spell format is correct in the Y-axis.

S3 Fig G: stretch out and draw back the figure as a fixed magnification ratio in order to consistent with others.
